# Vascular smooth muscle cell PRDM16 regulates circadian variation in blood pressure

**DOI:** 10.1172/JCI183409

**Published:** 2024-12-03

**Authors:** Zhenguo Wang, Wenjuan Mu, Juan Zhong, Ruiyan Xu, Yaozhong Liu, Guizhen Zhao, Yanhong Guo, Jifeng Zhang, Ida Surakka, Y. Eugene Chen, Lin Chang

**Affiliations:** 1 Department of Internal Medicine, Frankel Cardiovascular Center, University of Michigan, Ann Arbor, Michigan, USA.; 2 Institute of Cardiovascular Disease, Key Laboratory for Arteriosclerology of Hunan Province, Hunan International Scientific and Technological Cooperation Base of Arteriosclerotic Disease, Department of Pathophysiology, Hengyang Medical School, University of South China, Hengyang, Hunan, China.; 3 Department of Pharmacological and Pharmaceutical Sciences, University of Houston College of Pharmacy, Houston, Texas, USA.

**Keywords:** Vascular biology, Cardiovascular disease, Hypertension

## Abstract

Disruptions of blood pressure (BP) circadian variation are closely associated with an increased risk of cardiovascular disease. Thus, gaining insights into the molecular mechanisms of BP circadian variation is essential for comprehending BP regulation. Human genetic analyses suggest that PR domain–containing protein 16 (PRDM16), a transcription factor highly expressed in vascular smooth muscle cells (VSMCs), is significantly associated with BP-related traits. However, the roles of PRDM16 in BP regulation are largely unknown. Here, we demonstrate that BP in VSMC-specific *Prdm16*-KO (*Prdm16*^SMKO^) mice was significantly lower than that in control mice during the active period, resulting in aberrant BP circadian variation. Mesenteric artery rings from *Prdm16*^SMKO^ mice showed a reduced response to phenylephrine. Mechanistically, we identified adrenergic receptor α 1d (*Adra1d*) as a transcriptional target of PRDM16. Notably, PRDM16 exhibited a remarkable circadian expression pattern and regulated the expression of clock genes, particularly *Npas2*, which is crucial for BP circadian variation regulation. Consequently, PRDM16 deficiency in VSMCs caused disrupted BP circadian variation through a reduced response to adrenergic signaling and clock gene regulation. Our findings provide insights into the intricate molecular pathways that govern circadian fluctuations in BP.

## Abstract 2

The protein PRDM16, found in vascular muscle cells, helps regulate blood pressure’s daily fluctuations. Its absence leads to a non-dipping blood pressure pattern.

## Graphical abstract

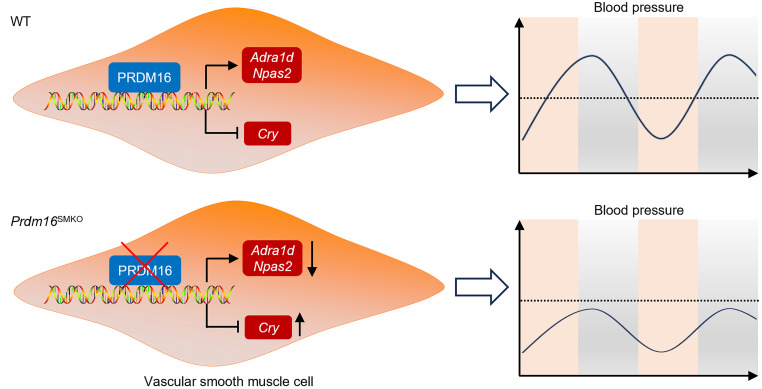

## Introduction

Blood pressure (BP) in humans has diurnal-nocturnal oscillations over a 24-hour cycle, known as the BP circadian rhythm, which is characterized by a morning surge and a nocturnal decrease (1). Declines in nocturnal BP can be classified into normal dippers (10%–20% drop), nondippers (0%–10% drop), extreme dippers (≥20% drop), and reverse dippers (negative percentage change in BP during sleep) (2). Abnormalities in BP circadian variations are closely linked to a higher risk of cardiovascular disease (CVD) (3, 4). Understanding the mechanisms behind BP circadian variation is important for hypertension management.

The contraction state of vascular smooth muscle cells (VSMCs) in blood vessels is critical for the regulation of BP levels (5). VSMC contractility is regulated by neurotransmitters, hormones, and vasoactive factors via GPCRs, leading to myosin light chain (MLC) phosphorylation and vascular contraction (6). The internal timing system, including the central clock in the hypothalamic suprachiasmatic nucleus (SCN), and peripheral clocks, with clock genes (*Bmal1*, *Clock*, *Per*, *Cry*), play a crucial role in BP regulation as well (7). Disruptions in clock genes such as *Bmal1*, *Npas2*, *Cry*, and *Clock* have been shown to change BP circadian rhythms (8, 9).

PR domain–containing protein 16 (PRDM16) is a transcriptional factor known for its role in brown adipocyte determination (10, 11). Recent findings have highlighted the crucial functions of PRDM16 in the cardiovascular system, including maintenance of normal heart development and function (12–17), sustainment of endothelial functions (18), and regulation of arterial development and vascular integrity (19). Importantly, PRDM16 is most highly expressed in the aorta and artery, and single-cell RNA-Seq (scRNA-Seq) analysis in aortas from both humans and mice revealed that PRDM16 is predominantly expressed in VSMCs (20), where it protects against abdominal aortic aneurysm (21). Recent genome-wide association studies (GWAS) and phenome-wide association studies (PheWAS) have linked several single nucleotide polymorphisms, either within or closest to the *PRDM16* gene, to BP-related traits, including diastolic BP (DBP), systolic BP (SBP), hypertension, coronary artery disease (CAD), and stroke (22–25). However, direct evidence of the role of PRDM16 in BP regulation is lacking. Using RNA-Seq analysis, we discovered that VSMC-specific *Prdm16*-KO mice (*Prdm16*^SMKO^) (21) exhibited dysregulated clock genes in the aorta, suggesting a potential link between PRDM16 in VSMCs and BP regulation. We thus hypothesized that PRDM16 in VSMC regulates BP circadian variation. In this study, we demonstrate that loss of function of PRDM16 in VSMCs caused nondipping BP as a result of reduced adrenergic receptor signaling and alterations in clock gene expression.

## Results

### PRDM16 expression in vascular cells and its association with BP-related traits.

**Figure 1:**
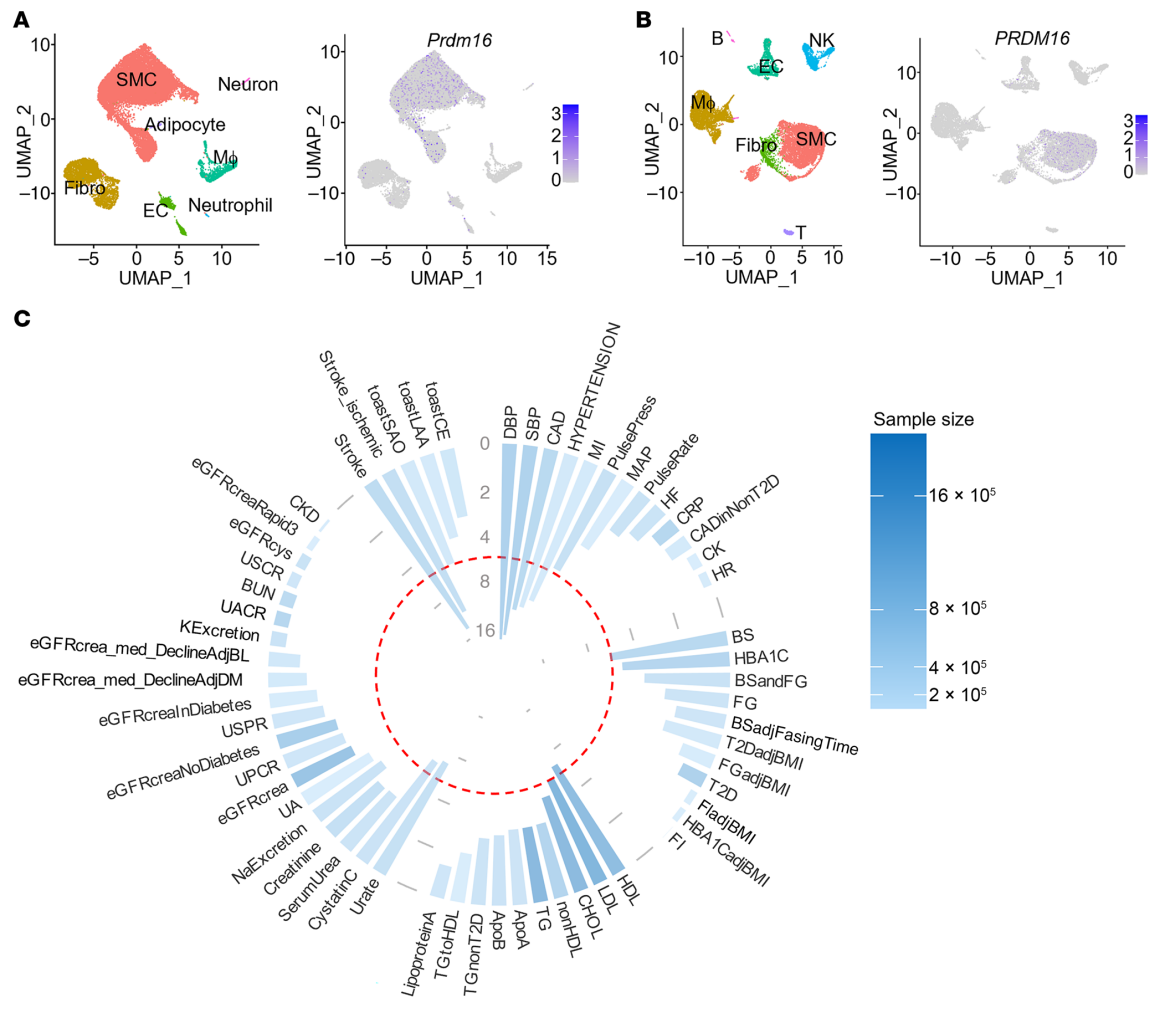
Predominant expression of PRDM16 in VSMCs and its gene-level associations with common variants. (**A** and **B**) Uniform manifold approximation and projection (UMAP) results showing higher expression of *Prdm16* in smooth muscle cells (SMCs) compared with other cell populations in the aorta and artery by scRNA-Seq analysis of mouse aorta (GEO GSE193265, a total of 22,980 cells were included in this analysis) (**A**) and human carotid artery (GEO GSE155468 and GEO GSE155512 were integrated for analysis, a total of 15,685 cells were included in this analysis) (**B**). Fibro, fibroblasts; EC, endothelial cells; Mϕ, macrophages; NK, NK cells; B, B cells; T, T cells. (**C**) Circular plot shows gene-level phenotypic associations for PRDM16. Data were sourced from the Common Metabolic Diseases Knowledge Portal (https://t2d.hugeamp.org/gene.html?gene=PRDM16), focusing on the “Common Variants Association Table” and “HuGE Scores Table” for group categorization. The data ancestry includes African American or Afro-Caribbean, African unspecified, Asian, European, Greater Middle Eastern, Hispanic or Latin American, and Sub-Saharan African. After filtering out phenotypes with sample sizes of 100,000 or fewer and keeping the cardiometabolic disease–related traits, 59 phenotypes across 5 groups were obtained. The circular plot organizes groups alphabetically and phenotypes within each group by *P* value. The *y* axis represents –log_10_-transformed *P* values for standardized comparison. A generally accepted threshold for significance of MAGMA results is *P* ≤ 2.5 × 10^–6^ (dashed red circle). The blue color represents the sample size. MI, myocardial infarction; MAP, mean arterial pressure; HF, heart failure; CRP, plasma C-reactive protein; CADinNonT2D, coronary artery disease in individuals without type 2 diabetes; CK, creatine kinase; HR, heart rate; BS, random glucose; HBA1C, hemoglobin A1C; BSandFG, random and fasting glucose; FG, fasting glucose; BSadjFastingTime, random glucose adj fasting time; T2DadjBMI, type 2 diabetes adj BMI; FGadjBMI, fasting glucose adj BMI; T2D, type 2 diabetes; FIadjBMI, fasting insulin adj BMI; HBA1CadjBMI, HBA1C adj BMI; FI, fasting insulin; CHOL, total cholesterol; nonHDL, non-HDL cholesterol; TG, triglycerides; ApoA, serum apolipoprotein A; ApoB, serum apolipoprotein B; TGnonT2D, triglyceride levels in individuals without type 2 diabetes; TGtoHDL, triglyceride-to-HDL ratio; Urage, serum urate; NaExcretion, urinary sodium excretion; UA, urinary albumin; eGFRcreat, serum creatinine; UPCR, urinary potassium-to-creatinine ratio; eGFRcreateNoDiabetes, eGFRcreat in individuals without diabetes; USPR, urinary sodium-to-potassium ratio; eGFRcreateInDiabetes, eGFRcreat in individuals with diabetes; eGFRcreat_med_DeclineAdjDM, eGFRcreat median annual decline adj diabetes status; eGFRcreat_med_DeclineAdjBL, eGFRcreat median annual decline adj baseline; KExcretion, urinary potassium excretion; UACR, urinary albumin-to-creatinine ratio; BUN, blood urea nitrogen; USCR, urinary sodium-to-creatinine ratio; eGFRcys, serum cystatin C; eGFRcreatRapid3, eGFRcreat decline, Rapid3 definition; CKD, chronic kidney disease; toastSAO, toast small artery occlusion; toastLAA, toast large artery atherosclerosis; toastCE, toast cardio-aortic embolism.

The Genotype-Tissue Expression (GTEx) Portal dataset indicates that *PRDM16* is predominantly expressed in the aorta and artery compared with other organs. We further validated this observation by quantitative PCR (qPCR) on multiple organs from mice (Supplemental Figure 1; supplemental material available online with this article; https://doi.org/10.1172/JCI183409DS1). The scRNA-Seq analysis revealed that PRDM16 was predominantly expressed in VSMCs in both mouse and human aortas (Figure 1, A and B). These data suggest that PRDM16 has crucial roles in vascular physiology and pathophysiology. Notably, GWAS and PheWAS have shown that the *PRDM16* gene–associated single nucleotide polymorphisms are significantly associated with BP-related traits, including DBP, SBP, hypertension, CAD, and any stroke (23, 24) (Figure 1C). However, the roles of PRDM16 in VSMCs in BP regulation is unknown.

### Loss of function of PRDM16 in VSMCs results in disrupted BP circadian variation.

**Figure 2:**
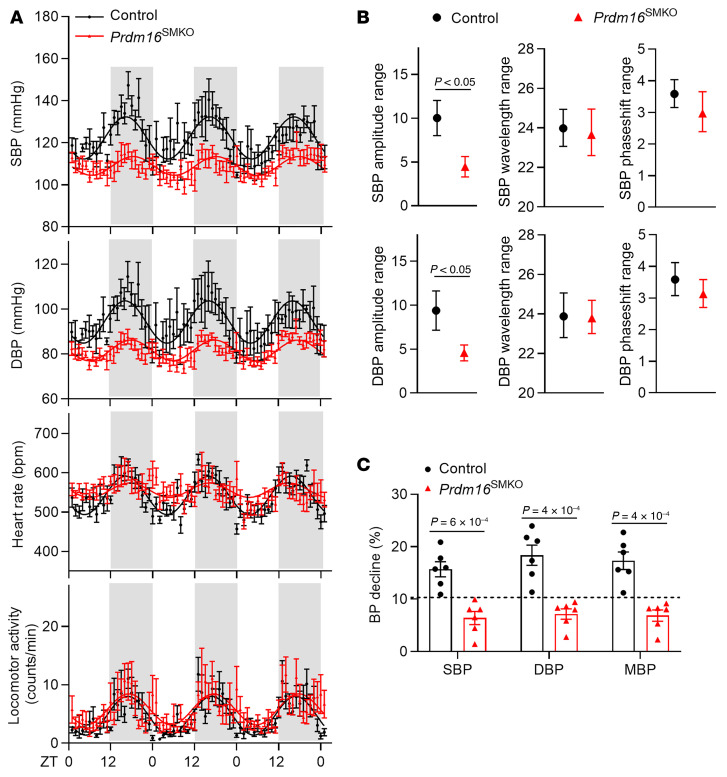
Loss of function of PRDM16 in VSMCs results in nondipping BP. (**A**) Radio telemetric measurements of SBP, DBP, HR, and locomotor activity in 16-week-old *Prdm16*^SMKO^ mice and control mice housed under normal conditions (12-hour light/12-hour dark cycle, 20°C–23°C) with free access to regular chow and water. ZT0 indicates lights on; ZT12 indicates lights off. Gray shadows indicate the nighttime. *n* = 6. (**B**) Characterization of SBP and DBP cycles, including amplitude range, wavelength range, and phase-shift range, were determined. *n* = 6. (**C**) Declines of SBP, DBP, and mean BP (MBP) in the resting phase relative to the active phase were analyzed. *n* = 6. Data in **A**–**C** are presented as the mean ± SEM. *P* values were determined by 2-tailed Student’s *t* test.

We monitored the ambulatory BP of male *Prdm16*^SMKO^ mice (the *SMMHC*-CreER^T2^ BAC transgene was inserted on the Y chromosome) (26) and control mice using radiotelemetry. Our findings revealed that during the active phase (lights-off period), basal BP (both SBP and DBP) in *Prdm16*^SMKO^ mice was markedly lower than that in control mice (Figure 2A). Interestingly, during the sleeping phase (lights-on period), we detected no significant difference in SBP and DBP between the 2 groups, although *Prdm16*^SMKO^ mice exhibited a tendency toward lower SBP and DBP (Figure 2A). Meanwhile, we observed a significant decrease in the amplitude range of both SBP and DBP in *Prdm16*^SMKO^ mice, whereas the circadian wavelength range and phase-shift range remained consistent when compared with those of control mice (Figure 2B). Consequently, BP decline from the active phase to the sleeping phase was less than 10%, indicating a nondipping BP pattern in *Prdm16*^SMKO^ mice (Figure 2C).

We further used a newly developed *Itga8*-CreER^T2^ (27) mouse model to delete PRDM16 in VSMCs. Female *Prdm16*^SMKO^ and control mice were subjected to radiotelemetric measurements. Consistent with the results from *SMMHC*-CreER^T2^–mediated KO of *Prdm16* in male mice, *Itga8*-CreER^T2^–mediated *Prdm16* KO in female mice also resulted in hypotension during the active phase and a nondipping BP pattern (Supplemental Figure 2, A–C).

The BP changes observed in *Prdm16*^SMKO^ mice were not attributable to their heart rate (HR), which was comparable between the 2 groups (Figure 2A and Supplemental Figure 2A). The cardiac function parameters such as ejection fraction (EF) and fractional shortening (FS) were also comparable between the 2 groups (Supplemental Tables 1 and 2). Furthermore, the 2 groups of mice exhibited similar levels of locomotor activity (Figure 2A and Supplemental Figure 2A) and whole-body metabolism. The energy expenditure, O_2_ consumption rate (VO_2_), and CO_2_ production rate (VCO_2_) did not show significant differences between *Prdm16*^SMKO^ and control mice (Supplemental Figure 3A). In alignment with these findings, the body weight, body composition (percentage of fat and lean mass), glucose tolerance, and insulin sensitivity were also similar across the groups (Supplemental Figure 3, B–E). Therefore, neither heart function nor whole-body metabolism appeared to cause the disrupted BP circadian variation observed in *Prdm16*^SMKO^ mice.

### PRDM16 deficiency in VSMC results in a reduced contractile response of resistance arteries to phenylephrine.

**Figure 3:**
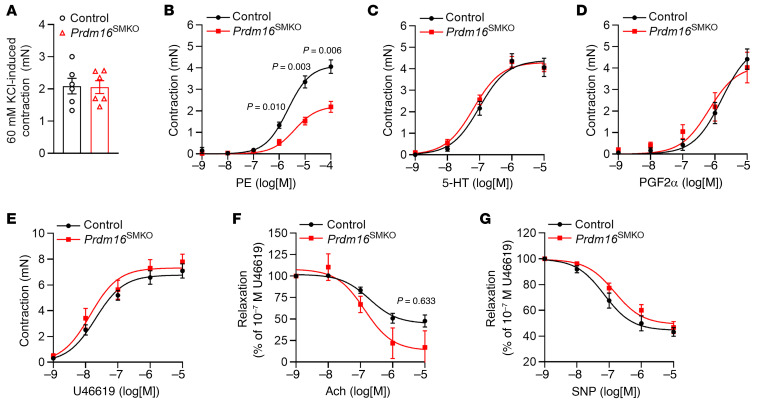
PRDM16 deficiency leads to a reduced contractile response to PE in mesenteric arteries. The first-order branches of superior mesenteric arteries were dissected from 10- to 12-week-old male *Prdm16*^SMKO^ mice and control mice. The vessels were cut into approximately 3 mm long rings, and wire myography was performed to determine the contraction force. (**A**) Aortic contraction induced by 60 mM KCl. (**B**–**E**) Concentration response curves were plotted for PE (**B**), 5-HT (**C**), PGF2α (**D**), and U46619 (**E**). (**F** and **G**) The vessels were first stimulated with U46619 to induce contraction, followed by treatment with Ach (**F**) and SNP (**G**), and concentration response curves were plotted. *n* = 6 mice per group. Data are presented as the mean ± SEM. *P* values were determined by 2-way ANOVA followed by Holm-Šidák multiple-comparison test.

We next investigated whether PRDM16 deletion in VSMCs affects the contractility of resistance arteries. We isolated the first-order branches of superior mesenteric arteries and determined their responses to various vasoconstrictors to discern the contribution of different mechanisms. We found that potassium chloride–induced (KCl-induced) contractions of the arterial rings were comparable between *Prdm16*^SMKO^ and control mice (2.05 ± 0.20 mN in *Prdm16*^SMKO^ mice vs. 2.09 ± 0.24 mN in control mice) (Figure 3A). However, arterial rings from *Prdm16*^SMKO^ mice showed a significantly decreased contractile response to phenylephrine (PE) compared with those from control mice (Figure 3B), while other vasoconstrictors including 5-hydroxytryptamine (5-HT, also known as serotonin), prostaglandin F2α (PGF2α), and U46619 (a thromboxane A2 agonist) induced similar responses in arterial rings isolated from *Prdm16*^SMKO^ and control mice (Figure 3, C–E). We also evaluated the vasorelaxation of arterial rings to acetylcholine (Ach) and sodium nitroprusside (SNP) and found no significant differences between *Prdm16*^SMKO^ and control mice (Figure 3, F and G).

### PRDM16 transcriptionally activates the expression of the adrenoceptor Adra1d.

**Figure 4:**
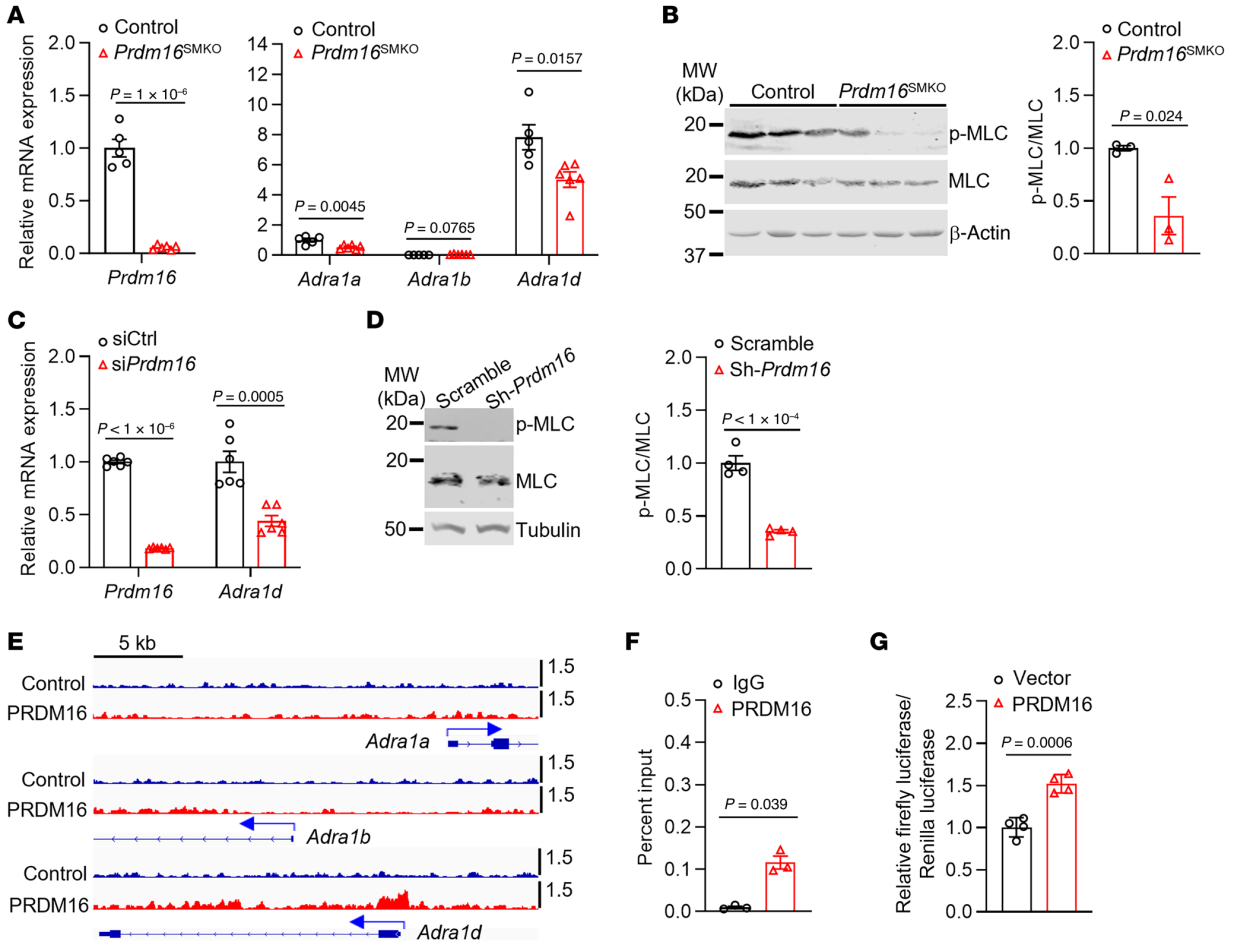
PRDM16 transcriptionally regulates *Adra1d* expression and adrenoceptor signaling. (**A**) The abdominal aorta was isolated from 12-week-old male *Prdm16*^SMKO^ mice and control mice, and mRNA expression levels of *Prdm16* and adrenoceptor α 1a family members, including *Adra1a*, *Adra1b*, and *Adra1d*, were determined by qPCR. *n* = 5 control mice, *n* = 6 *Prdm16*^SMKO^ mice. (**B**) The thoracic aorta was isolated from 12-week-old male *Prdm16*^SMKO^ mice and control mice, and MLC20 phosphorylation levels (p-MLC) induced by PE for 3 minutes were determined by Western blotting. Each lane represents pooled lysates from 1–2 mice. MW, molecular weight. (**C**) Primary VSMCs isolated from rat thoracic aortas were transfected with control siRNA (siCtrl) (10 nM) or si*Prdm16* (10 nM) for 48 hours, and relative expression levels of *Adra1d* were determined by qPCR. *n* = 6. (**D**) Representative Western blot gels showing decreased MLC20 phosphorylation levels in cultured VSMCs upon *Prdm16* KD by shRNA. *n* = 4. (**E**) IGV tracks of PRDM16 ChIP-Seq in primary mouse fibroblasts showing that the *Adra1d* promoter region contains PRDM16-binding peaks. (**F**) ChIP-qPCR analysis showing PRDM16 binding to the promoter region of *Adra1d* in primary mouse VSMCs. *n* = 3. (**G**) Luciferase assay in NIH/3T3 cells transfected with *Adra1d* promoter–driven luciferase reporters and PRDM16 expression plasmids. *n* = 4. Data are presented as the mean ± SEM. *P* values were determined by 2-tailed Student’s *t* test.

To further explore the underlying mechanisms mediating the impaired contractile response specifically to PE in *Prdm16*^SMKO^ mice, we performed qPCR to determine the mRNA expression levels of the related receptors for the above-mentioned vasoconstrictors. PE selectively binds to and activates adrenoceptor α 1 receptors, which consists of adrenoceptor α 1a (encoded by *Adra1a*), adrenoceptor α 1b (encoded by *Adra1b*), and adrenoceptor α 1d (encoded by *Adra1d*). *Adra1d* is the most highly expressed α1-adrenergic receptor in the media, while *Adra1a* is primarily expressed in the adventitia, and *Adra1b* shows negligible expression levels (Supplemental Figure 4A). We found that the mRNA expression of *Adra1a* and *Adra1d* in the aorta was significantly reduced in *Prdm16*^SMKO^ mice compared with control mice (Figure 4A). Consistent with the comparable responses to 5-HT, PGF2α, U46619, Ach, and SNP in artery rings between *Prdm16*^SMKO^ and control mice, the mRNA expression levels of serotonin receptor 2A (*Htr2a*), prostaglandin F receptor (*Ptgfr*), thromboxane A2 receptor (*Tbxa2r*), and nitric oxide synthase 3 (*Nos3*) in the aorta were comparable between *Prdm16*^SMKO^ and control mice (Supplemental Figure 4B). It is well established that adrenoceptor activation leads to phosphorylation of the downstream effector MLC, which is critical for vascular contraction (6). We found that phosphorylation of MLC in the aorta of *Prdm16*^SMKO^ mice was significantly decreased compared with that in aortas of control mice (Figure 4B). We further validated that both the mRNA expression levels of *Adra1d* (Figure 4C) and the phosphorylation of MLC (Figure 4D) in cultured VSMCs were markedly reduced upon *Prdm16* knockdown (KD).

Considering that PRDM16 is a transcriptional regulator (11), we next performed ChIP-Seq analysis. We identified a binding peak of PRDM16 in the promoter region of *Adra1d* but not of *Adra1a* or *Adra1b* (Figure 4E). In addition, ChIP-qPCR analysis further confirmed the binding of PRDM16 in the promoter region of *Adra1d* (Figure 4F). To investigate whether the binding of PRDM16 to the *Adra1d* promoter regulates its activity, we cloned the promoter surrounding the binding peak region of *Adra1d* to a luciferase reporter vector (pGL4.10) and transfected the reporter vector into NIH/3T3 cells. We found that coexpression of PRDM16 significantly activated the expression of luciferase (Figure 4G). All these data demonstrate that PRDM16 transcriptionally activated the expression of the adrenoceptor *Adra1d*, which mediated VSMC contraction induced by PE. PRDM16 deficiency in VSMCs resulted in reduced *Adra1d* expression and a blunted response to the adrenoceptor signaling pathway, particularly during the active phase in which the sympathetic nervous system is active. This led to nondipping BP.

### Loss of the VSMC contractile phenotype upon PRDM16 ablation.

**Figure 5:**
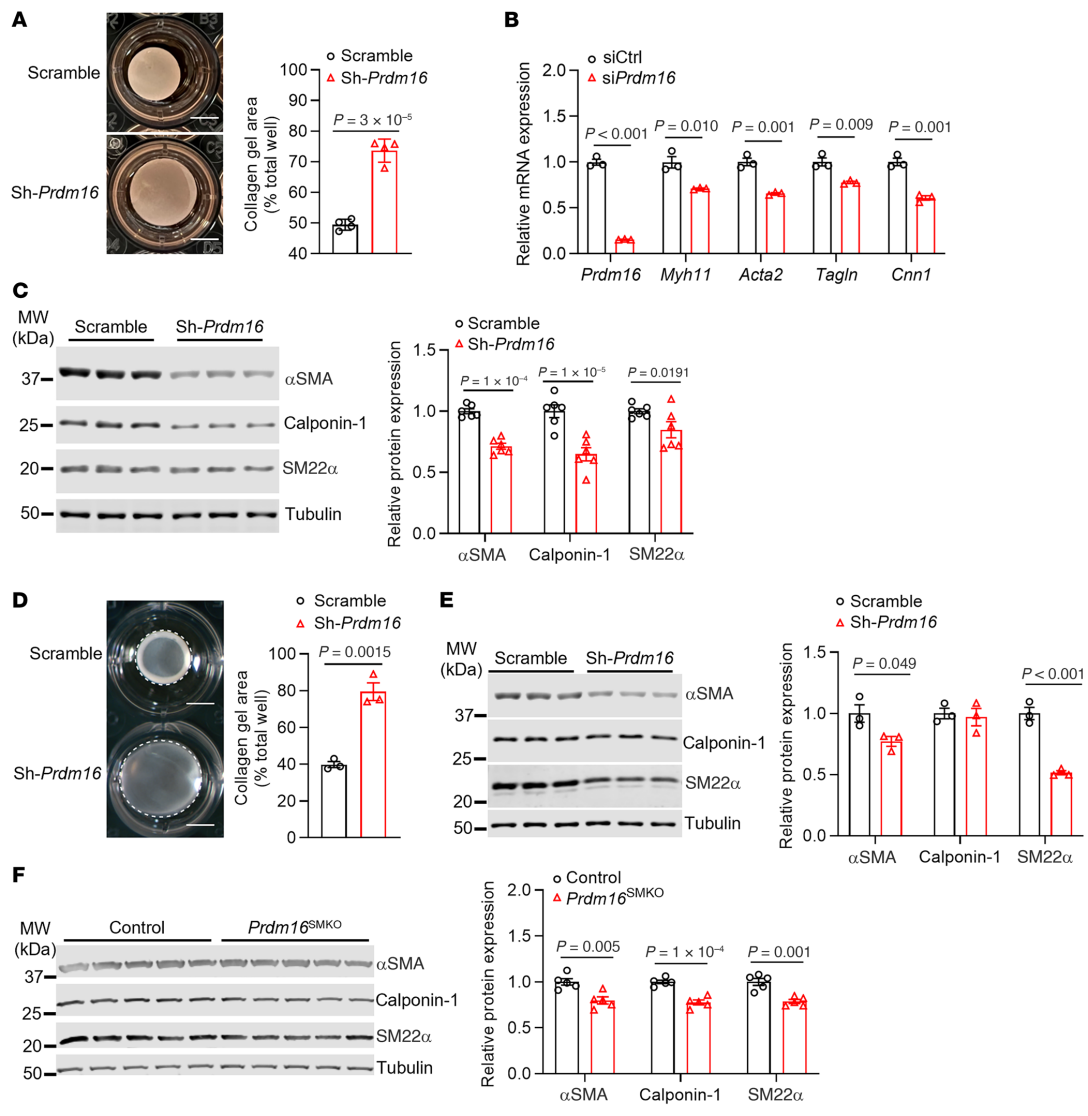
PRDM16 is essential for VSMC contractility. (**A**–**C**) Primary VSMCs isolated from rat thoracic aortas were infected with lentivirus carrying shRNA (**A** and **C**) or transfected with control siRNA (siCtrl) (10 nM) or si*Prdm16* (10 nM) (**B**) for 48 hours. Cells with stable shRNA expression were selected by puromycin. (**A**) Representative images of the collagen-based contraction assay and quantitative analysis. *n* = 4. Scale bars: 0.5 cm. (**B**) Relative expression levels of contractile genes were determined by qPCR. *n* = 3. (**C**) Relative protein expression levels were determined by Western blotting; representative gels are shown. *n* = 6. (**D**) Representative image of collagen-based contraction assay of cultured VSMCs isolated from rat mesenteric arteries upon *Prdm16* KD by shRNA. *n* = 3. Scale bars: 0.5 cm. (**E**) Relative protein expression levels in cultured VSMCs isolated from rat mesenteric arteries were determined by Western blotting upon *Prdm16* KD by shRNA. *n* = 3. (**F**) Expression of contractile markers in the medial layer of thoracic aorta were determined by Western blotting. *n* = 5. Data are presented as the mean ± SEM. *P* values were determined by 2-tailed Student’s *t* test.

Our previous study showed that the mRNA levels of *Acta2* (encodes smooth muscle cell α actin [αSMA]) were decreased in the aorta of *Prdm16*^SMKO^ mice compared with levels in control mice (21). To further explore the functional role of PRDM16 in contractile phenotype, we performed a 3D, collagen-based cell contraction assay, which revealed that KD of *Prdm16* dramatically impeded the contraction of VSMCs (Figure 5A). We consistently found that *Prdm16* KD markedly reduced mRNA levels of all 4 recognized VSMC marker genes, namely *Myh11* (encodes smooth muscle myosin heavy chain, also known as MYH11), *Acta2*, *Tagln* (encodes smooth muscle protein 22α [SM22α]), and *Cnn1* (encodes calponin 1) (Figure 5B), and protein expression of αSMA, calponin 1, and SM22α (Figure 5C). We also used primary VSMCs isolated from rat mesenteric arteries to further confirm that PRDM16 was essential for maintaining the VSMC contractile phenotype in resistance arteries (Figure 5, D and E). Furthermore, we observed a significant reduction in protein expression of αSMA, calponin 1, and SM22α in the medial layer of thoracic aortas from *Prdm16*^SMKO^ mice compared with control mice (Figure 5F). However, the involvement of PRDM16 in controlling contractile marker gene expression may not have resulted from direct transcriptional regulation, since no binding peaks were found in the promoter regions of these genes (Supplemental Figure 5). Taken together, these data indicate that PRDM16 was essential for maintaining the contractile phenotype of VSMCs. Loss of function of PRDM16 led to impaired VSMC contraction.

### Circadian expression of Prdm16 and its regulation of the expression of canonical clock genes in VSMCs.

**Figure 6:**
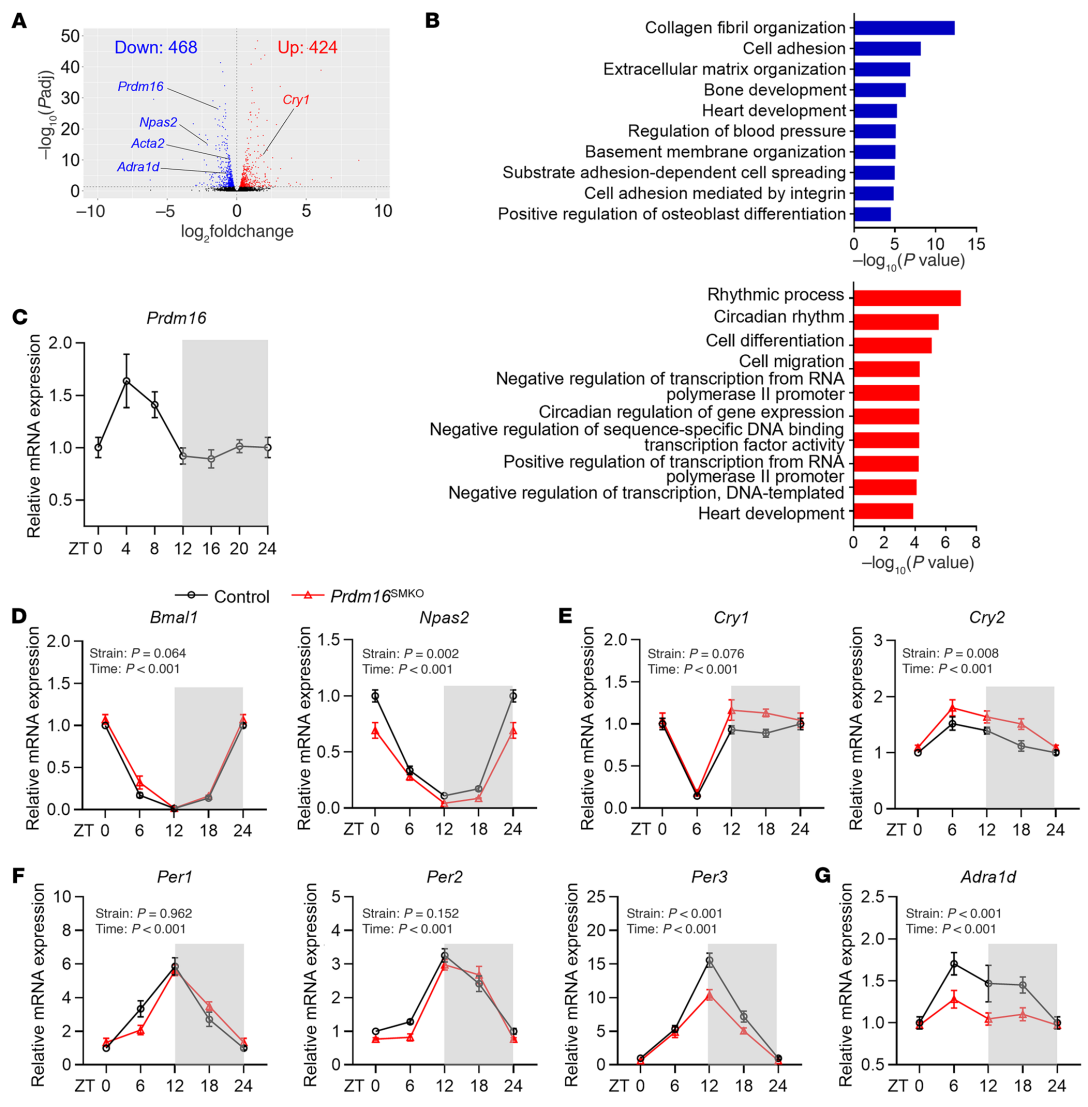
PRDM16 is involved in circadian rhythm regulation. (**A**) Volcano plot of the DEGs in thoracic aortas from *Prdm16*^SMKO^ and control mice (*n* = 3). Upregulated DEGs are highlighted in red; downregulated DEGs are highlighted in blue. (**B**) The DEGs were analyzed for GO biological process (GO_BP) enrichment using the Database for Annotation, Visualization, and Integrated Discovery (DAVID), and the top 10 significantly enriched terms are shown. Red bars and blue bars indicate GO_BP results from upregulated DEGs and downregulated DEGs, respectively. (**C**) Thoracic aortas from 10-week-old male C57/BL6J mice were harvested at 4-hour intervals over a 24-hour period. *Prdm16* mRNA expression was determined by qPCR. ZT0 indicates lights on; ZT12 indicates lights off. *n* = 6 per time point. (**D**–**G**) The thoracic aortas from 10-week-old male *Prdm16*^SMKO^ mice and control mice were harvested at 6-hour intervals over a 24-hour period. mRNA expression of canonical clock genes, including (**D**) *Bmal1* and *Npas2*, (**E**) *Cry1* and *Cry2*, and (**F**) *Per1*, *Per2*, and *Per3*, and the (**G**) PRDM16 transcriptional target gene *Adra1d*, were determined by qPCR. *n* = 5–6 per group per time point. Gray shadows in **C**–**G** indicate the nighttime. Data are presented as the mean ± SEM. *P* values were determined by 2-way ANOVA followed by Holm-Šidák multiple-comparison test.

The CpG sites for PRDM16 show notable hypomethylation in the placental tissue of night shift workers (28), suggesting that PRDM16 is linked to circadian rhythms. RNA-Seq analysis of the aorta indicated that the genes with differential expression between *Prdm16*^SMKO^ and control mice were enriched in the regulation of BP and the circadian rhythm (Figure 6 A and B). We thus determined the 24-hour mRNA expression of *Prdm16* in the aorta and found that *Prdm16* mRNA levels in the aorta were higher during the resting phase and lower during the active phase (Figure 6C). We also examined the expression patterns of clock genes, including *Bmal1*, *Npas2*, *Cry1*, *Cry2*, *Per1*, *Per2*, and *Per3*, in the aorta of *Prdm16*^SMKO^ mice and their control littermates. We found that the *Npas2* mRNA levels in the aorta of *Prdm16*^SMKO^ mice were lower than those in the control mice at all time points (Figure 6D). The *Cry1/Cry2* mRNA levels in the aorta of *Prdm16*^SMKO^ mice were higher than those in control mice during light-off period (Figure 6E), whereas peak mRNA levels of *Per3* at Zeitgeber time 12 (ZT12) were lower in *Prdm16*^SMKO^ mice (Figure 6F). These data suggest that PRDM16 may control the expressions of clock genes. In addition, we found that the circadian expression pattern of *Adra1d* was almost abolished in *Prdm16*^SMKO^ mice (Figure 6G).

**Figure 7:**
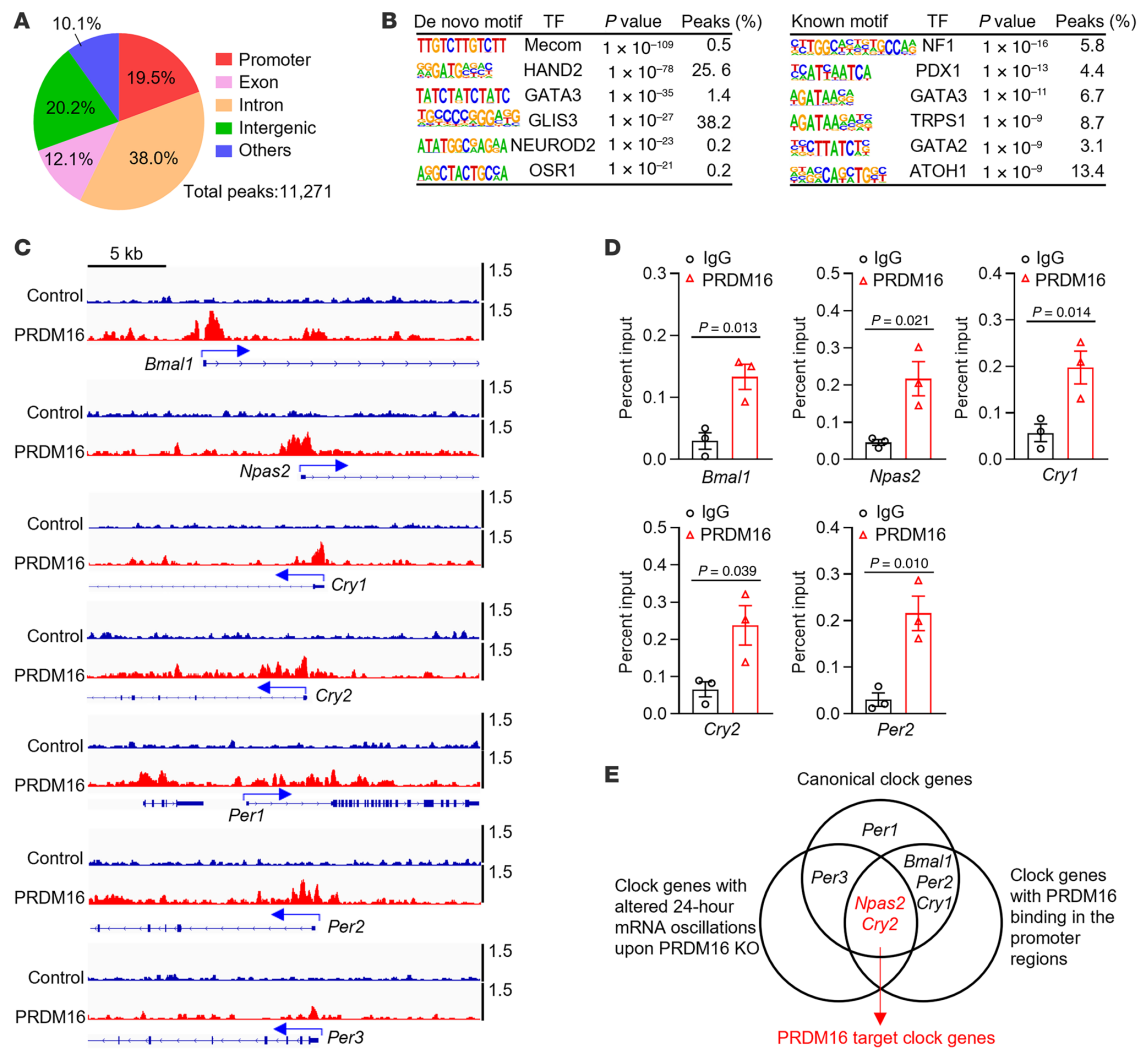
PRDM16 regulates the expression of canonical circadian genes. (**A**) Genomic distribution of PRDM16-binding sites from PRDM16 ChIP-Seq analysis. (**B**) HOMER motif analysis of PRDM16-binding sites. The top 6 transcription factors are shown. (**C** and **D**) IGV tracks (**C**) and ChIP-qPCR analysis (**D**) showing PRDM16 binding to the promoter regions of canonical clock genes. *n* = 3. (**E**) Venn diagram showing PRDM16-targeted clock genes. Data are presented as the mean ± SEM. *P* values were determined by 2-tailed Student’s *t* test (**D**).

From our ChIP-Seq analysis, we identified 11,271 peaks representing putative PRDM16-binding sites across the genome, with 19.5% residing in the promoter, 12.1% in exons, 38.0% in introns, and 20.2% in intergenic regions (Figure 7A). Motif analysis of PRDM16 ChIP-Seq peaks revealed that consensus DNA binding motifs for MECOM (also known as PRDM3), which has a binding motif almost identical to that for PRDM16 (12), and several other transcription factors including HAND2 and GATA family transcription factors were highly enriched (Figure 7B). Notably, we found PRDM16-binding peaks in the promoter regions of *Bmal1*, *Npas2*, *Cry1*, *Cry2*, and *Per2* (Figure 7C). Using ChIP-qPCR analyses, we confirmed that PRDM16 bound to the promoter regions of *Bmal1*, *Npas2*, *Cry1*, *Cry2*, and *Per2* (Figure 7D). We next developed a strategy of comparing clock genes with both altered 24-hour mRNA oscillations upon PRDM16 KO (Figure 6, D–F), PRDM16 binding signals in the promoter region (Figure 7C), and ChIP-qPCR (Figure 7D), and identified *Npas2* and *Cry2* as PRDM16 target clock genes (Figure 7E). In addition, we verified that PRDM16 expression significantly activated *Npas2* promoter–driven luciferase expression (Supplemental Figure 6). Although the underlying mechanisms are yet to be fully addressed, *Npas2^mut^* mice (with decreased expression of *Npas2*) are hypotensive (8), and *Cry*-null mice are hypertensive upon salt induction (9), these results are consistent with the hypotensive phenotype observed in *Prdm16*^SMKO^ mice. The gene ontology (GO) analysis of differentially expressed genes (DEGs) with PRDM16 binding at their promoters revealed that the upregulated target genes were significantly enriched in the rhythmic process and the circadian rhythm (Supplemental Figure 7 and Supplemental Table 3), further emphasizing the involvement of PRDM16 in the regulation of clock genes.

### Interactions between the two PRDM16 targets: clock genes and Adra1d.

**Figure 8:**
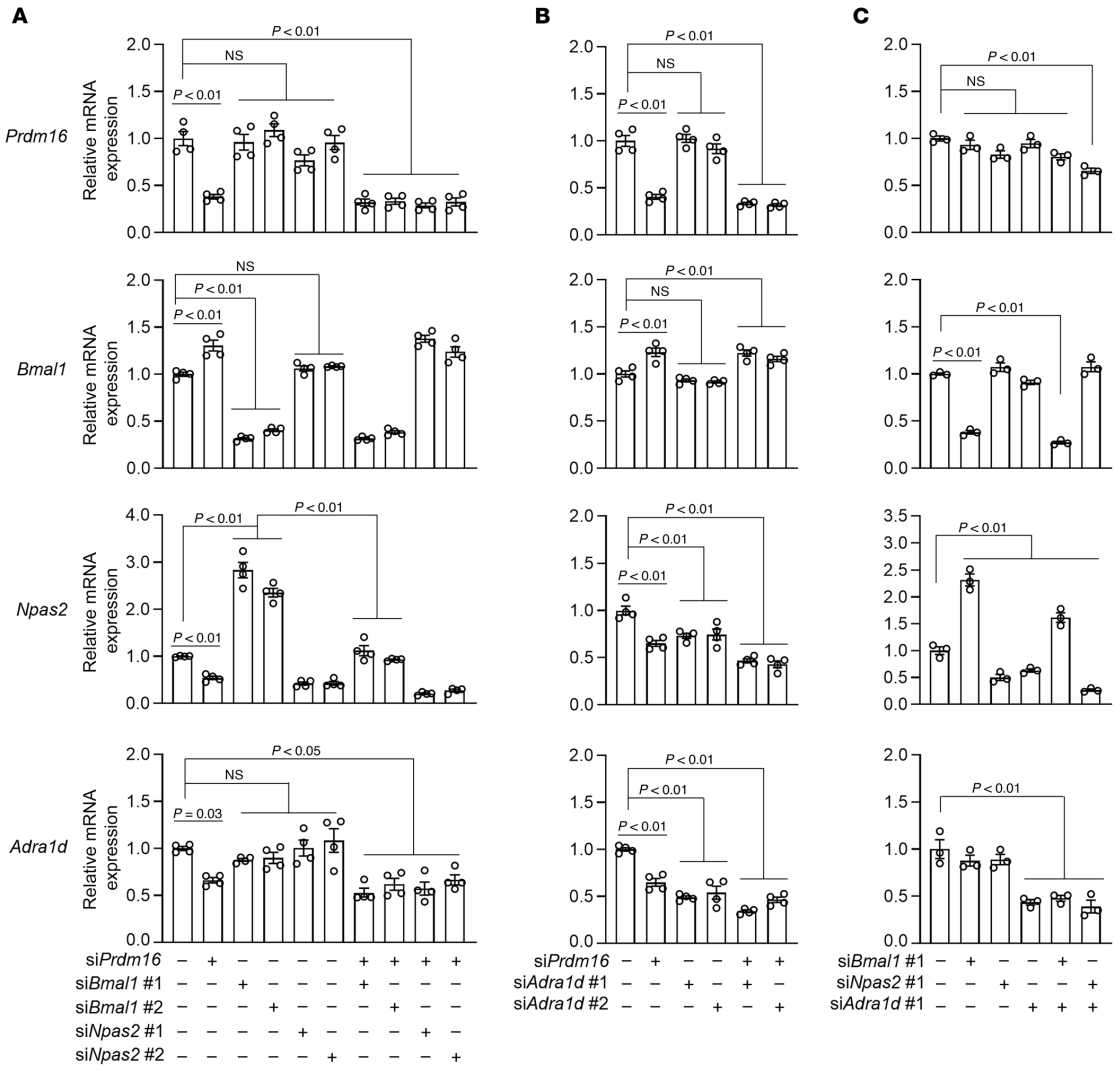
Interactions among *Prdm16*, *Adra1d*, and *Npas2*:*Bmal1*. (**A**–**C**) Primary VSMCs isolated from rat thoracic aortas were transfected with the indicated siRNA targeting *Prdm16*, *Bmal1*, *Npas2*, and *Adra1d* (total amounts of siRNA were balanced by the control siRNA) for 48 hours, and expression levels of the indicated genes were determined by qPCR. *n* = 3–4. Data are presented as the mean ± SEM. *P* values were determined by 1-way ANOVA followed by Holm-Šidák multiple-comparison test.

We next investigated how these 3 factors, PRDM16, clock genes (primarily NPAS2 and its binding partner, BMAL1), and ADRα1d, collaboratively regulate the BP circadian variation. For this purpose, we first asked whether NPAS2:BMAL1 regulates *Prdm16* and *Adra1d* expression. As shown in Figure 8A, KD of *Npas2* or *Bmal1* had no effect on *Prdm16* and *Adra1d* expression in primary VSMCs. Interestingly, KD of *Bmal1* resulted in a dramatic increase in *Npas2* expression, which could be a compensatory response, as previously indicated (29). Next, we investigated whether ADRα1d could regulate *Prdm16* and *Npas2* or *Bmal1* expression. As shown in Figure 8B, *Adra1d* KD did not influence *Prdm16* or *Bmal1* expression but markedly reduced *Npas2* expression. It is worth noting that *Adra1d* and *Prdm16* double-KD synergistically decreased *Npas2* expression, indicating that the regulation of *Npas2* by ADRα1d and PRDM16 likely occurs through different pathways. Neither *Npas2*/*Bmal1* nor *Adra1d* KD affected *Prdm16* expression (Figure 8C). Taken together, we found that PRDM16 played a dominant role in regulating the expression of *Adra1d* and clock genes, whereas ADRα1d independently regulated *Npas2* expression.

In summary, these data indicated that PRDM16 is a critical factor that regulates BP levels and variation by targeting the expression of *Adra1d* and clock genes. These findings extensively expand the critical roles of PRDM16 in the cardiovascular system and provide the translational basis for human studies.

## Discussion

GWAS and PheWAS have identified several single nucleotide polymorphisms, either within or closest to the *PRDM16* gene, that are closely related to BP and CVD (22–24, 30). In this study, we provide the first evidence to our knowledge that PRDM16 in VSMCs regulates BP circadian variation by regulating the expression of *Adra1d* and core clock genes in VSMCs. Loss of PRDM16 function in mouse VSMCs resulted in an impaired BP circadian variation, with lower BP during the active phase.

The contractile function of VSMCs in blood vessels is critical for the regulation of vascular tone and BP levels. PRDM16 has been identified as a single-nucleus assay for transposase-accessible chromatin with high-throughput sequencing (snATAC-Seq) SMC marker gene, showing a positive correlation with traditional SMC markers and a negative correlation with fibromyocyte marker genes (22). In our study, we demonstrated that PRDM16 deficiency in VSMCs markedly reduced the expression of contractile marker genes and impaired collagen-based contraction in vitro. Interestingly, our ChIP-Seq analysis revealed that PRDM16 did not directly bind to the promoter regions of these contractile marker genes, indicating that its regulatory effects were likely mediated through indirect mechanisms. One potential pathway involves the TGF-β signaling cascade. Previous research has shown that PRDM16 can negatively regulate *Tgfb2*, a key upstream ligand of the TGF-β pathway, possibly by binding to the promoter region of *Tgfb2* (20). Additionally, in gastric cancer cells, PRDM16 has been found to inhibit TGF-β signaling by interacting with Ski oncogene, a known repressor of TGF-β signaling (31). These findings suggest a model in which PRDM16 indirectly modulates contractile gene expression in VSMCs by altering TGF-β signaling dynamics. The TGF-β signaling pathway is well known for its pleiotropic regulatory effects on VSMC phenotype and function. Maintaining homeostasis within VSMCs requires a balanced activation of TGF-β signaling (neither deficient nor excessive activation is beneficial). Thus, PRDM16 in VMSCs may indirectly affect the expression of contractile marker genes and the contraction of blood vessels by modulating TGF-β signaling.

Sympathetic hormones, including epinephrine and norepinephrine, induce VSMC contraction by binding to α1-adrenergic receptors. Our data indicate that PRDM16 bound to the promoter region and transcriptionally activated *Adra1d* expression in VSMCs, although *Adra1a* expression was also reduced in the arteries of *Prdm16*^SMKO^ mice. However, our ChIP-Seq analysis did not reveal any PRDM16 binding peaks in the promoter region of *Adra1a*, implying that the reduced *Adra1a* mRNA expression may have been a secondary effect of *Adra1d* downregulation. Other important vasoconstriction factors such as angiotensin II (Ang II) (32) may not contribute to PRDM16-mediated vascular tone regulation. Expression levels of the Ang II receptor *Agtr1a* in the aorta were not different between *Prdm16*^SMKO^ mice and control mice (Supplemental Figure 4B). Therefore, these findings collectively suggest that PRDM16 played a crucial role in BP regulation by modulating the expression of α1-adrenergic receptors, particularly *Adra1d*. Consistent with our data, a previous study showed that *Adra1d* expression was decreased in the right ventricle of cardiomyocyte-specific *Prdm16*-KO mice (12). The cardiac-null mutation of *Prdm16* causes hypotension in female mice (33). As a result, mice lacking PRDM16 function in VSMCs exhibit lower BP during the active phase due to a blunted response to sympathetic stimulation, resulting in a blunted BP elevation.

Additionally, previous studies documented that clock genes regulate vascular tone and BP circadian variation by modulating both catecholamine production and responsiveness through adrenergic receptors in the aorta. For instance, global KO of *Bmal1* or *Npas2* mutation in mice reduces BP, especially during the active phase (8). *Bmal1* deletion reduces plasma norepinephrine and epinephrine levels and increases the expression of catechol-*O*-methyltransferase (*Comt*), an enzyme responsible for catecholamine clearance, in the aorta (8). Similarly, endothelium-specific *Bmal1* deletion reduces BP during the active phase without altering plasma catecholamines (34). VSMC-specific *Bmal1* deletion also reduces active-phase BP, although the plasma catecholamine levels and adrenoceptor expression levels in VSMCs remain unclear (35). However, expression of the *Cry* gene is regulated by *Bmal1*, and *Cry* plays a role in circadian BP regulation by modulating α-adrenergic receptor–mediated vasoconstriction. Global *Cry*–deficient mice show diminished catecholamine-mediated vasoconstriction due to reduced α-adrenoceptor expression in the aorta (9). In addition to the crosstalk between clock genes in α-adrenergic receptor signaling, the regulation of BP variation by clock genes and *Adra1d* may occur through different pathways. BMAL1 directly activates *Rock2* expression in VSMCs, regulating MLC phosphorylation and vascular contraction through a Ca^2+^-independent pathway (35). In contrast, ADRα1d signals through Ca^2+^-dependent pathways involving the Gq/11 family of G proteins, leading to MLC phosphorylation, actin polymerization, and vascular contraction (6). Moreover, *Bmal1* KO compensatorily increased *Npas2* expression in the aorta (8), which might be a compensatory way to correct the disrupted clock. And *Npas2* is a target gene of PRDM16 (36). In this study, we comprehensively explored the relationship between PRDM16, clock genes, and the newly identified PRDM16 target gene *Adra1d*. PRDM16 functions as a master regulator that governs the expression of both *Npas2* and *Adra1d*. On the one hand, ADRα1d responds to sympathetic nervous system activation by inducing adrenergic receptor signaling pathway transduction to trigger Ca^2+^-dependent vascular contraction. On the other hand, the NPAS2:BMAL1 complex functions in a Ca^2+^-independent manner (or via other pathways yet to be defined) to induce vascular contraction. These 2 arms thus collaboratively result in blunted BP elevation in the active phase in *Prdm16*^SMKO^ mice.

Consistent with expression in adipose tissues and whole eyes (37, 38), our data showed that PRDM16 expression in the aorta had a circadian pattern. However, little is known about the upstream regulator of PRDM16. Previous studies have suggested that BMAL1 might regulate PRDM16, yet conclusions vary across different tissues. For example, in *Bmal1*-KO mice, *Prdm16* expression is significantly reduced in the olfactory bulb (39), whereas it is markedly increased in brown adipocytes differentiated from preadipocytes (40, 41). It has also been shown that *Per3*-KO mice have reduced *Prdm16* expression in whole eyes (38), and the homozygous staggerer mutant mouse, which carries a deletion within the nuclear receptor ROR alpha gene, shows increased *Prdm16* expression in inguinal and epididymal adipose tissue (37). In our study, we did not observe any differences in *Prdm16* mRNA expression upon *Bmal1* or *Npas2* KD in cultured VSMCs. Additionally, PRDM16 has been shown to undergo posttranslational modifications (PTMs), including acetylation, polyubiquitination, and SUMOylation, which modulate its protein stability and functions (42–44). Notably, S-nitrosylation is an oxidative PTM that a nitric oxide (•NO) group is reversibly added to cysteine residues (45). The production of endogenous •NO by NOS varies according to the circadian rhythm (46). S-nitrosylation of proteins has been linked to the regulation of vascular tone and BP (47, 48). Through computational tools such as GPS-SNO (49) and iSNO-PseAAC (50), it has been predicted that PRDM16 can undergo S-nitrosylation at the Cys587 residue (51). Therefore, PRDM16 might regulate BP circadian variations through its NO-dependent S-nitrosylation, which needs further studies in the future.

In summary, our research elucidates a previously unknown mechanism by which PRDM16 in VSMCs regulates BP circadian variation. This finding has critical implications for understanding the pathogenesis of hypertension and related cardiovascular disorders. By linking PRDM16 to the regulation of clock genes and α-adrenergic receptor signaling, our study identifies a potential target for therapeutic interventions aimed at restoring normal BP circadian rhythms. Such treatments could be particularly beneficial for patients with hypertension, or sleep apnea and for those who work night shifts, all of whom commonly experience disrupted BP patterns. Furthermore, targeting PRDM16 might offer a new avenue for personalized medicine. The identification of PRDM16-linked single nucleotide polymorphisms associated with BP traits suggests that genetic screening could help identify individuals at higher risk for circadian BP disruptions and related cardiovascular events. Early intervention in these high-risk populations could improve clinical outcomes and reduce the burden of CVD. In conclusion, our study not only advances the understanding of BP regulation but also opens new pathways for translational research and clinical applications aimed at mitigating hypertension and its consequences.

## Methods

### Sex as a biological variable.

Our study examined male and female animals, and similar findings are reported for both sexes.

### Animals.

Male VSMC-specific *Prdm16*-KO (*Prdm16*^SMKO^) mice were obtained by crossbreeding *Prdm16*^lox/lox^ floxed mice (strain number 024992, The Jackson Laboratory) (52) with *Myh11*-CreER^T2^ mice (strain number 019079, The Jackson Laboratory) (26), and female *Prdm16*^SMKO^ mice were generated by crossbreeding *Prdm16*^lox/lox^ mice with *Itga8*-CreER^T2^ mice (27). All mice were maintained on a C57BL/6J background and housed under regular housing conditions (12-hour light/12-hour dark cycle, 20–23°C) with free access to regular chow diet and water. To induce *Prdm16* KO, 8-week-old mice were treated with tamoxifen (75 mg/kg/day, catalog T5648, MilliporeSigma) in corn oil (catalog C8267, MilliporeSigma) for 5 consecutive days by oral gavage, followed by a 2-week washout period. *Prdm16*^lox/lox^ mice and *Myh11*-CreER^T2^ or *Itga8*-CreER^T2^ mice were used as a control.

### Radiotelemetric measurement of BP.

BP was measured by radiotelemetry (HD-X11 or PA-C10, Data Sciences International), as described previously (29). In brief,10- to 12-week-old mice were anesthetized with 2% isoflurane inhalation. The left common carotid artery was cannulated with the telemetry catheter, and the telemetry transmitter was secured in the abdominal cavity. Following recovery from surgery for at least 10 days, SBP, DBP, HR, and locomotor activity were continuously recorded.

### Echocardiography in mice.

In brief,10- to 12-week-old male mice were anesthetized with 2% isoflurane inhalation. The hair on the chest area was removed using hair removal cream. A generous amount of ultrasound gel was applied to the chest area and the high-frequency transducer was positioned on the chest area at the left parasternal area to obtain standard cardiac views, including long-axis views, short-axis views, and M-mode imaging for the measurements of cardiac function parameters such as ejection fraction (EF), fractional shortening (FS), stroke volume, and cardiac output.

### Glucose tolerance test and insulin tolerance test.

For the glucose tolerance test (GTT), mice were fasted for 6 hours. Glucose in the drinking water was administered by oral gavage at a dose of 2 g/kg BW. Blood samples were collected through tail vein puncture at 30-minute intervals, and blood glucose levels were measured until they returned to normal levels. For the insulin tolerance test (ITT), mice were administered an i.p. injection of insulin at a dose of 100 U/kg BW. Blood samples were collected via tail vein puncture, and blood glucose levels were measured at 30-minute intervals until they returned to normal levels.

### Whole-body metabolism measurement by the Promethion system (indirect calorimetry).

Mice were acclimated to a metabolic chamber, where they were housed individually under controlled environmental conditions, including 24°C environment temperature, 37% humidity, and a 12-hour/12-hour light/dark cycle. The airflow rate through the chambers was adjusted at a level that would maintain the oxygen differential around 0.3% at resting conditions. The oxygen consumption (VO_2_) and carbon dioxide production (VCO_2_) were sampled for 5 seconds at 10-minute intervals for the calculation of total energy expenditure (EE) and the respiratory exchange ratio (RER).

### Wire myography.

The first-order branches of the superior mesenteric arteries were dissected from 10- to 12-week-old male mice and rinsed in ice-cold physiological saline solution (PSS) (130 mM NaCl, 4.7 mM KCl, 1.18 mM KH_2_PO_4_, 1.17 mM MgSO_4_, 0.026 mM EDTA, 14.9 mM NaHCO_3_, 5.5 mM glucose, and 1.6 mM CaCl_2_). After removal of perivascular adipose tissue, the arteries were cut into approximately 3 mm long rings and mounted onto a wire myograph chamber (610 M, Danish Myo Technology A/S) containing 8 mL PSS aerated with a mixture of 95% O_2_ and 5% CO_2_ and maintained at 37°C. The vessel rings were then allowed to rest for 20 minutes and stretched to the optimal basal tension (~1.5 mN) in a stepwise manner as previously described (29), followed by 3 consecutive KCl (60 mM in PSS) responses for wakeup. After equilibration for 30 minutes, the cumulative concentration responses to PE (catalog P6126, MilliporeSigma, stock solution 0.1 M in Milli-Q water), 5-HT (catalog H9523, MilliporeSigma, stock solution 0.1 M in Milli-Q water), PGF2α (catalog P5069, MilliporeSigma, stock solution 10 μM in ethanol), and U-46619 (catalog 16450, Cayman Chemical) were assessed to characterize vasoconstriction. To characterize vasodilation, the vessel rings were preconstricted with U-46619, and then the cumulative dose responses to Ach (catalog A2661, MilliporeSigma, stock solution 0.1 M in Milli-Q water) and SNP (catalog 71778, MilliporeSigma, stock solution 0.1 M in Milli-Q water) were determined. Constriction was measured as tension and expressed in absolute millinewton (mN) values.

### Cell culturing.

Primary VSMCs were isolated from the thoracic aorta of 10-week-old male rats as previously described (21) and cultured in DMEM/F12 (catalog 11330032, Gibco, Thermo Fisher Scientific) supplemented with 10% FBS (catalog 10438026, Gibco, Thermo Fisher Scientific). Primary VSMCs were cultured at 37°C, 5% CO_2_ in a humidified cell culture incubator and used from passages 3–10 in all experiments. For the KD assay, primary VSMCs were transfected with control siRNA (siCtrl) (10 nM) or si*Prdm16* (10 nM) using Lipofectamine RNAiMAX (catalog 13778150, Invitrogen, Thermo Fisher Scientific), according to the manufacturer’s protocols. Forty-eight to 72 hours later, RNA and protein samples were harvested. siCtrl, *Prdm16* siRNA (target sequence: UGACAGUUUAGCCGGGAAA), *Bmal1* siRNA 1 (target sequence: GAUCACGACUAAUUGCUAU), *Bmal1* siRNA 2 (target sequence: AGACUGGACUUCCGGUUAA), *Npas2* siRNA 1 (target sequence: UAGGAUACCUGCCCUUUGA), *Npas2* siRNA 2 (target sequence: CACCAUGACUUCCGGUAUA), and *Adra1d* siRNA (catalog MQ-091737-01-0002) were obtained from Dharmacon, Horizon Discovery.

### qPCR analysis.

qPCR was performed as previously described (21). Briefly, total RNA was extracted using the RNeasy Mini kit (for cells, catalog 74106, QIAGEN) or RNeasy Micro kit (for aortic tissues, catalog 74004, QIAGEN), followed by cDNA synthesis with the SuperScript III First-Strand Synthesis System (catalog 18080, Life Technologies, Thermo Fisher Scientific). Relative mRNA expression was calculated by normalization to *Gapdh* levels using the 2^–ΔΔCt^ method. Primer sequences used in this study are listed in Supplemental Table 4.

### Western blot analysis.

Protein lysates from cultured VSMCs or the medial layer of thoracic aorta were prepared with RIPA buffer (catalog 89901, Thermo Fisher Scientific) supplemented with protease inhibitor cocktail (catalog 11873580001, Roche) and were resolved by SDS-PAGE and transferred onto nitrocellulose membranes (catalog 1620115, Bio-Rad). The membranes were blocked in 5% nonfat dry milk in TBS with 0.1% Tween 20 detergent (TBS-T) for 1 hour at room temperature and probed with the following primary antibodies overnight at 4°C: anti-αSMA (1:1,000; catalog MA5-11547, Invitrogen, Thermo Fisher Scientific), anti–calponin 1 (1:1,000; catalog ab46794, Abcam), anti-transgelin (1:1,000; catalog ab46794, Abcam), anti-tubulin (1:1,000; catalog 2148, Cell Signaling Technology), and anti–β-actin (1:2,000; catalog 3700, Cell Signaling Technology). The signals were captured and quantified using Image Studio, version 3.1 (Odyssey CLx).

To determine the phosphorylation levels of MLC, thoracic aorta was dissected and equilibrated in PSS for 20 minutes at 37°C. Thirty minutes after 3 consecutive KCl (60 mM in PSS) responses for wakeup, the thoracic aorta was treated with vehicle and PE (0.1 mM) for 3 minutes. The tissues were then snap-frozen in liquid N_2_ and homogenized in RIPA buffer supplemented with protease inhibitor cocktail and phosphatase inhibitor cocktail 3 (catalog P0044, MilliporeSigma). Following sonication (Branson Sonifier SLPe, 15 seconds of 35% amplification, 3 times), the lysates were cleared by centrifugation for 2 minutes at 10,000*g* at 4°C. The protein concentrations of the supernatants were determined using Pierce BCA protein assay kits (catalog 23227, Thermo Fisher Scientific). Equal amounts of protein lysates were analyzed by Western blotting as described above. The following primary antibodies were used: anti–phosphorylated MLC2 (Ser19) (1:1,000; catalog 3671, Cell Signaling Technology) and anti-MLC2 (1:1,000; catalog 3672, Cell Signaling Technology).

### Luciferase assay.

The promoter region was amplified with PCR and subcloned into the pGL4.11[luc2P] vector (catalog E6661, Promega) using the In-Fusion HD Cloning Plus Kit (catalog 638910, TaKara Bio). All PCR products were validated by Sanger sequencing. The luciferase plasmids along with the PRDM16 expression vector or empty controls were then cotransfected into NIH/3T3 cells (catalog CRL-1658, American Type Culture Collection [ATCC]) using Lipofectamine 2000 (catalog 11668019, Thermo Fisher Scientific) according to the manufacturer’s instructions. Twenty-four hours after transfection, cells were harvested in passive lysis buffer, and luciferase activity was determined by dual luciferase assay (catalog E1910, Promega). Data are presented as the relative luciferase activity against *Renilla* activity.

### Collagen-based contraction assay.

The collagen-based contraction assay was performed as described previously (53). In brief, 2.3 mg/mL collagen (catalog A1048301, Gibco, Thermo Fisher Scientific) solution was prepared according to the manufacturer’s protocols and was mixed with the cell suspension (3 × 10^6^ cells/mL) at a volume ratio of 4:1. The collagen-cell mixture was then seeded into a 24-well plate and incubated for 30 minutes at 37°C, followed by addition of 0.5 mL Opti-MEM I to the gel lattice. After 24 hours, the gel was released, and its size was measured and analyzed using ImageJ software (NIH).

### RNA-Seq analysis.

RNA samples from the thoracic aorta of mice with a RNA integrity number (RIN) of greater than 7 were submitted to the Advanced Genomics Core of the University of Michigan for RNA-Seq, as we previously described (54).

### ChIP assay.

A ChIP assay was performed with the SimpleChIP Enzymatic Chromatin IP Kit (catalog 9003, Cell Signaling Technology), according to the manufacturer’s instructions. Briefly, mouse fibroblasts were infected with lentivirus carrying PRDM16 or vector (as the control) for 48 hours and were selected with 2 μg/mL puromycin for 1 week. Cells were then incubated with 1% paraformaldehyde (catalog 158127, MilliporeSigma) for 10 minutes at room temperature to crosslink proteins with DNA, followed by neutralization with glycine for 5 minutes at room temperature. The nuclei were prepared and digested with Micrococcal Nuclease for 20 minutes at 37°C, followed by sonication (Branson Sonifier SLPe, 20 seconds of 35% amplification, 3 times). The purified sheared chromatin was immunoprecipitated with anti-Flag antibody (1 μg, catalog 14793, Cell Signaling Technology), anti-PRDM16 antibody (2 μg, catalog ab106410, Abcam), or an equal amount of normal IgG (catalog 2729, Cell Signaling Technology) overnight at 4°C with gentle rotation. The precipitated DNA was extensively washed with low-salt buffer and high-salt buffer, and the eluted protein-DNA complexes were reversed with proteinase K for 2 hours at 65°C. Purified DNA was used for qPCR analysis. ChIP-qPCR data are expressed as a percentage of the input, which was calculated using the following equation: [percentage input = 2% × 2^[Ct(2% input sample] – Ct[IP sample])^].

### ChIP-Seq.

Purified DNA as described above was subjected to ChIP-Seq as previously described (55). The DNA library preparation and sequencing were performed by the Advanced Genomics Core of the University of Michigan. Briefly, the DNA library was prepared with the NEB Next Ultra II DNA Library Prep Kit for Illumina (New England Biolabs [NEB]) and sequenced on a NovaSeq 6000 platform (Illumina) to generate pair-end 101 bp reads. An average of 25 million paired reads was generated for each sample (IgG and anti-Flag groups). The reads were mapped to the mouse genome (NCBI GRCm38) with bowtie2. The peak calling was done with MACS2 (with the following settings: -f BAMPE –g hs –B --SPMR –q 0.01). Peaks were aligned to the nearest transcription start site (TSS) with HOMER, version 4.11 (http://homer.ucsd.edu/homer/). The peaks were visualized using the Integrative Genomics Viewer (IGV).

### Statistics.

Statistical analyses were performed using GraphPad Prism 10.3.1 (GraphPad Software). All data were assessed for variance and normality. The sample size was determined on the basis of preliminary studies and our previous publications (21, 29). A 2-tailed Student’s *t* test was used for comparisons between 2 groups. A 1-way or 2-way ANOVA followed by the Holm-Šidák multiple-comparison test was used for comparisons among 3 or more groups, as specified in the figure legends. A *P* value of less than 0.05 was considered significant. The data are presented as the mean ± SEM.

### Study approval.

All animal study protocols were approved by the IACUC of the University of Michigan.

### Data availability.

All data generated in this study are included in this manuscript (and its supplemental material). All data used to generate graphs are provided in the Supporting Data Values file. RNA-Seq and ChIP-Seq data are available in the NCBI’s Gene Expression Omnibus (GEO) database (GEO GSE272891). Additional detailed information is available from the corresponding author upon request.

## Author contributions

ZW and LC contributed to the design of the research studies and conducted experiments and acquired and analyzed data. WM, J Zhong, RX, YG, and J Zhang contributed to critical discussions and interpretation of the data and revision of the manuscript. YL, GZ, and IS contributed to analysis of the RNA-Seq data and plotting of the human genetic analyses results. YC and LC contributed to study conceptualization, supervision, project administration, and funding acquisition. ZW and LC wrote the manuscript.

## Supplementary Material

Supplemental data

Unedited blot and gel images

Supporting data values

## Version 1 12/03/2024

In-Press Preview

## Version 2 02/03/2025

Electronic publication
