# Supplementary material for: Vascular smooth muscle cell PRDM16 regulates circadian variation in blood pressure
Source: J Clin Invest. 2024 Dec 3;135(3):e183409. doi: 10.1172/JCI183409 (PMC11785921; doi:10.1172/JCI183409)
Supplement: Supplemental data [file jci-135-183409-s111.pdf]

Vascular smooth muscle cell PRDM16 regulates circadian variation in blood pressure

Zhenguo Wang,[1] Wenjuan Mu,[1] Juan Zhong,[1] Ruiyan Xu,[1,2] Yaozhong Liu,[1] Guizhen Zhao,[1,3] Yanhong Guo,[1] Jifeng Zhang,[1] Ida Surakka,[1] Y. Eugene Chen,[1] and Lin Chang[1]

[1]Department of Internal Medicine, Frankel Cardiovascular Center, University of Michigan, Ann Arbor, MI, USA

[2]Institute of Cardiovascular Disease, Key Laboratory for Arteriosclerology of Hunan Province, Hunan International Scientific and Technological Cooperation Base of Arteriosclerotic Disease, Department of Pathophysiology, Hengyang Medical School, University of South China, Hengyang, Hunan, China

[3]Department of Pharmacological and Pharmaceutical Sciences, University of Houston College of Pharmacy, Houston, TX, USA

Supplemental Figures 1 to 7

Supplemental Tables 1 to 4

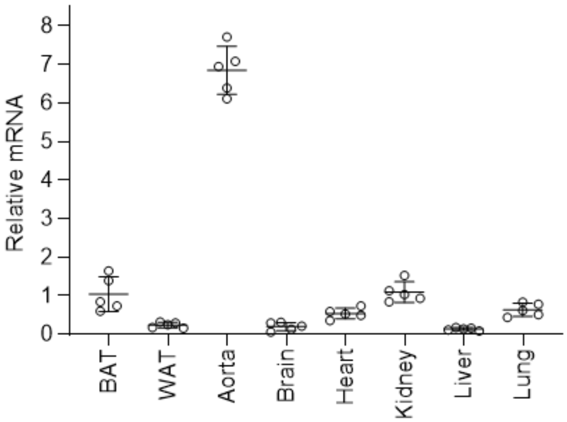

Supplemental Figure 1. PRDM16 is predominantly expressed in the aorta and artery in mice. Data from GTEx Portal (dbGaP Accession phs000424.v8.p2) indicates the highest PRDM16 expression in the aorta and artery, validated by qPCR analysis across multiple tissues of C57BL/6J mice. The relative *Prdm16* mRNA in brown adipose tissue (BAT) was set as 1. WAT, mesenteric white adipose tissue. Data are shown as mean ± SD. n = 5.

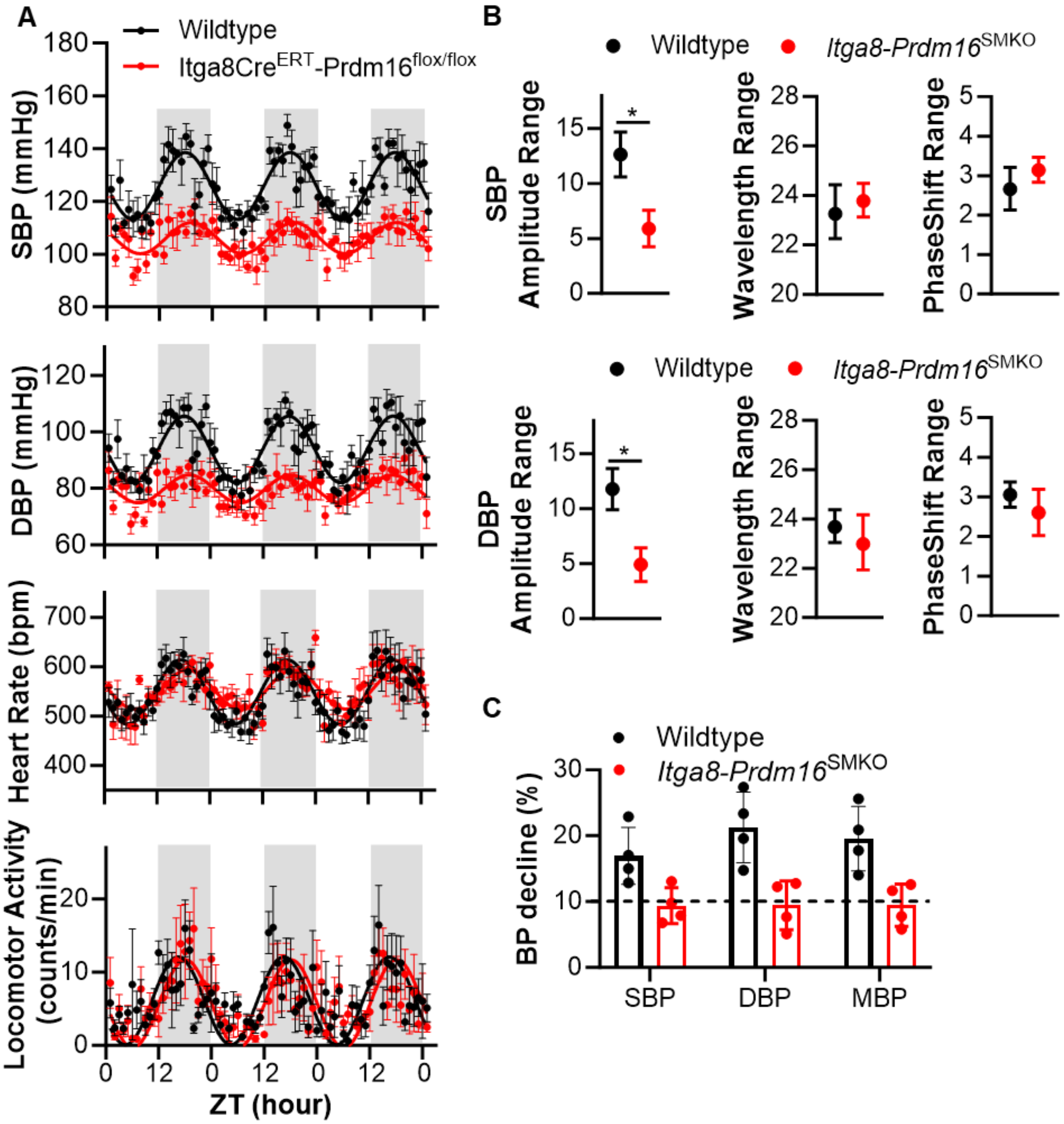

Supplemental Figure 2. PRDM16 deficiency in female VSMC results in hypotension during active phase. (**A**) Radiotelemetry measurement of systolic blood pressure (SBP), diastolic blood pressure (DBP), heart rate, and locomotor activity in 16-week-old *Prdm16*^{SMKO} mice and control mice housed under normal conditions (12-hour light/dark cycle, 20-23°C) with free access to regular chow diet and water. n = 4. (**B**) Characterization of SBP and DBP cycles, including amplitude range, wavelength range, and phaseshift range, were determined. n = 4. (**C**) Declines of SBP, DBP, and mean BP (MBP) in the resting phase relative to the active phase were analyzed. n = 4. Data in (**A**-**C**) are presented as mean ± SEM. *p* values were determined by 2-tailed Student's *t* test. *$p < 0.05$.

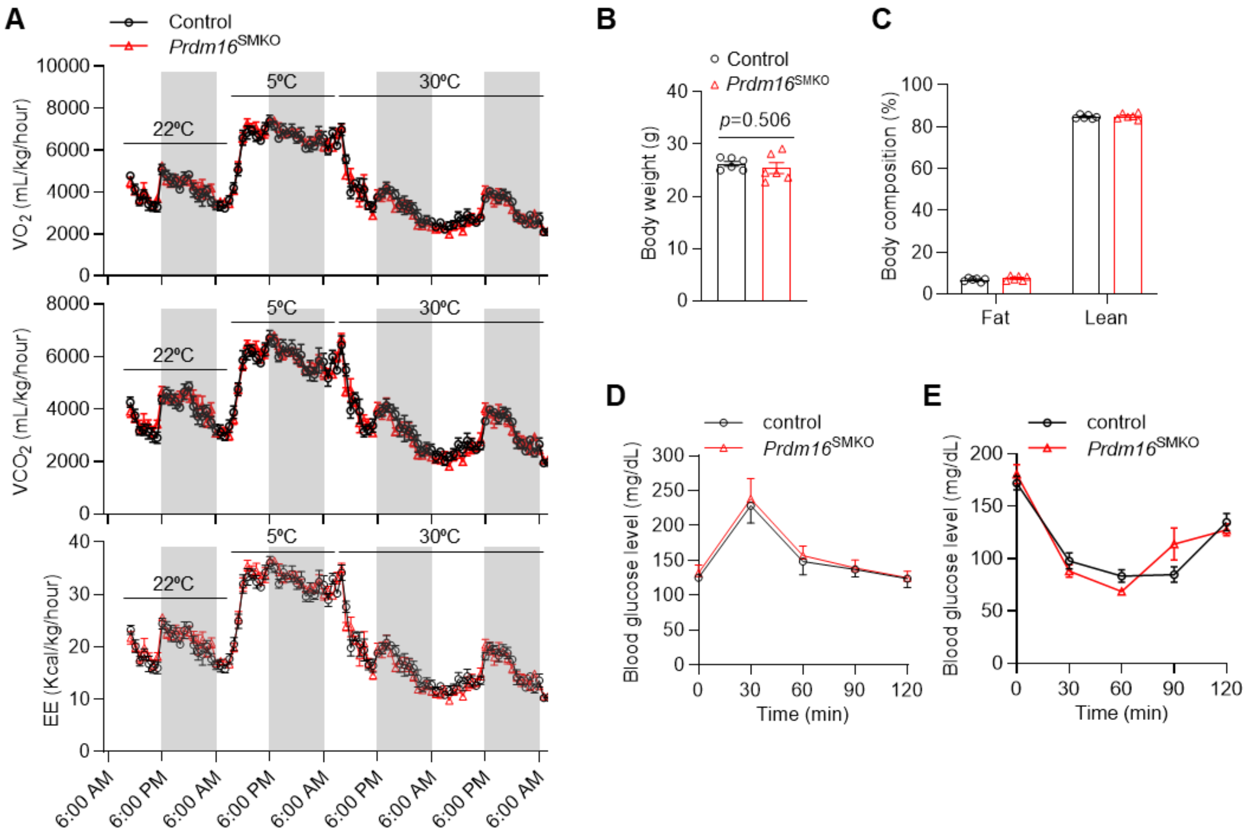

Supplemental Figure 3. Whole-body metabolism was not altered by PRDM16 deficiency in VSMC. (**A**) Twelve-week-old male mice were acclimated to single housing for one week and the O_2 consumption rate (VO_2) and CO_2 production rate (VCO_2) were recorded by the Promethion System. The energy expenditure (EE) was calculated based on VO_2 and VCO_2. The housing temperature was indicated in the figure. n = 6. (**B**) Body weight. (**C**) Body composition. (**D**) Oral glucose tolerance test (2 g/kg glucose). (**E**) Intraperitoneal insulin tolerance test (1.5 U/kg insulin). n = 6. Data are presented as mean ± SEM. *p* values were determined by 2-tailed Student's *t* test (for **B** and **C**) or two-way ANOVA followed by Holm-Šidák multiple-comparison test (for **D** and **E**).

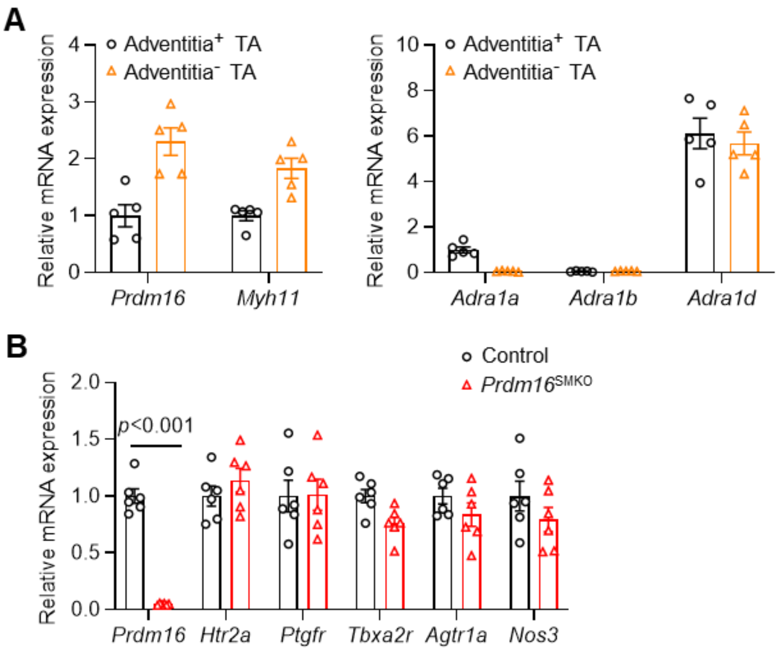

Supplemental Figure 4. Effects of PRDM16 on contractility-related signaling pathways. (**A**) The thoracic aorta (TA) was dissected from 10-12-week-old male C57BL/6J mice, followed by digestion with Type 2 Collagenase (1.5 mg/mL in DMEM/F12 1:1 medium) at 37°C for 5 minutes, the adventitia was carefully removed and the remained tissue was snap frozen in liquid N_2. The gene expression was determined by qPCR. The relative expression of *Adra1a* was set as 1 and serves as control. n = 5. (**B**) The mRNA expression of serotonin receptor 2A (*Htr2a*), prostaglandin F receptor (*Ptgfr*) and thromboxane A2 receptor (*Tbxa2r*), angiotensin II receptor, type 1a (*Agtr1a*), and nitric oxide synthase 3 (*Nos3*) in the aorta were determined by qPCR. n = 6. Data are presented as mean ± SEM. *p* values were determined by 2-tailed Student's *t* test.

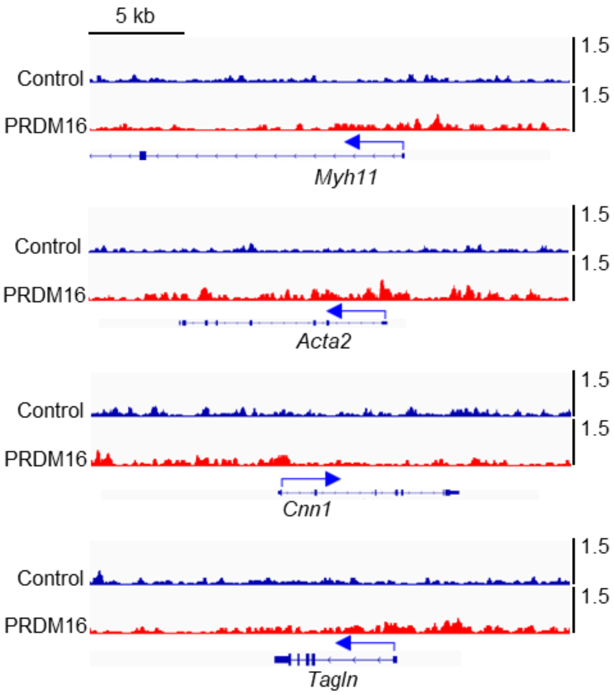

Supplemental Figure 5. IGV tracks of the VSMC marker genes. PRDM16 ChIP-seq in primary mouse fibroblast cells showing no PRDM16 binding peaks in the promoter regions of *Myh11*, *Acta2*, *Cnn1*, and *Tagln*.

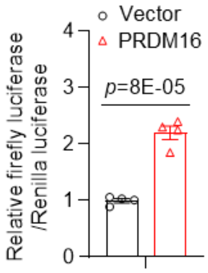

Supplemental Figure 6. PRDM16 regulates *Npas2* promoter-driven luciferase expression. Luciferase assay in NIH/3T3 cells transfected with *Npas2* promoter-driven luciferase reporters and PRDM16 expression plasmids. n = 4. Data are presented as mean ± SEM. *p* values were determined by 2-tailed Student's *t* test.

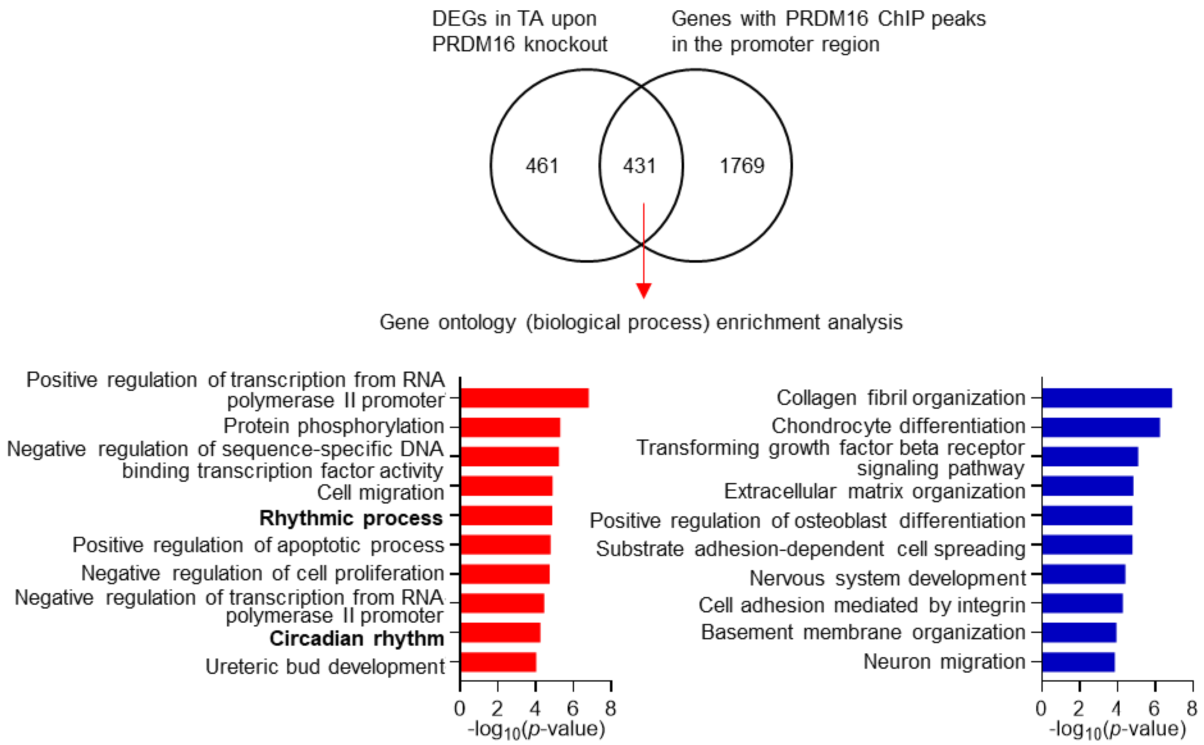

Supplemental Figure 7. GO analysis of DEGs with PRDM16 binding peaks in their promoter regions. The DEGs in Figure 6A with PRDM16-binding sites in their promoter regions were analyzed for gene ontology biological process (GO_BP) enrichment using DAVID, and the top 10 significantly enriched terms are shown. Red bars and blue bars indicate GO_BP results from upregulated DEGs and downregulated DEGs, respectively.

Supplemental Table 1. Echocardiogram (M-mode short axis)

	IVSd (mm)	IVSs (mm)	LVIDd (mm)	LVIDs (mm)	LVPWd (mm)	LVPWs (mm)	FS (%)	EF (%)
Control	0.77±0.13	1.12±0.10	3.64±0.26	2.45±0.28	0.58±0.07	0.89±0.09	33±4	62±5
Prdm16^{SMKO}	0.70±0.17	1.05±0.22	3.74±0.26	2.62±0.22	0.66±0.09	0.91±0.11	30±2	58±4

IVSd, interventricular septum (diastole); IVSs, interventricular septum (systole); LVIDd, left ventricular internal diameter (diastole); LVIDs, left ventricular internal diameter (systole); LVPWd: left ventricular posterior wall (diastole); LVPWs: left ventricular posterior wall (systole); FS: left ventricular Fractional Shortening; EF: left ventricular ejection fraction. n = 6. Data are presented as mean ± SD.

Supplemental Table 2. Echocardiogram (B-mode long axis)

	LV area d (mm^2)	LV area s (mm^2)	CO (mL/min)	SV (μL)	LV vol d (μL)	LV vol s (μL)
Control	20.63±2.36	11.60±1.89	13.32±2.98	30.56±4.23	52.84±9.81	22.28±5.63
Prdm16^{SMKO}	21.62±2.17	13.64±1.25	12.90±2.12	31.14±4.70	51.64±13.71	25.29±3.84

LV area d, the area defined by the inner wall of the LV in diastole parasternal long axis; LV area s, the area defined by the inner wall of the LV in systole parasternal long axis; CO, cardiac output of the LV; SV, stroke volume; LV vol d, LV volume in diastole; LV vol s, LV volume in systole.

Supplemental Table 3. Differentially expressed genes (DEGs) showing PRDM16 binding peaks in their promoter regions

Gene	Chr	Start	End	Distance _to_TSS	baseMean	log2FC	padj	Regulation
Npas2	chr1	39193836	39194282	-213	115.18	-2.14	9.8E-14	Down
Arntl	chr7	113207579	113208644	600	38.08	-1.75	8.6E-05	Down
Cilp2	chr8	69887823	69888133	-586	56.65	-1.64	4.7E-04	Down
Rasl11a	chr5	146844952	146845710	260	81.75	-1.46	2.1E-07	Down
Slc4a8	chr15	100761922	100762321	374	165.04	-1.36	7.7E-04	Down
Mmp28	chr11	83462665	83463003	206	173.33	-1.32	2.2E-08	Down
Nr4a1	chr15	101266129	101266529	-517	1875.77	-1.28	5.4E-03	Down
Lox	chr18	52529231	52529588	312	2296.38	-1.24	1.7E-09	Down
Tet1	chr10	62886817	62887094	369	301.15	-1.22	6.8E-10	Down
Srgap3	chr6	112948638	112948905	-1505	578.55	-1.18	2.1E-05	Down
Nos1ap	chr1	170589448	170590033	109	176.03	-1.10	8.3E-07	Down
Txlnb	chr10	17794189	17794441	-1904	160.71	-1.08	9.2E-10	Down
Hspb7	chr4	141421634	141421902	989	4447.43	-1.02	1.7E-20	Down
Foxo6	chr4	120287153	120287676	-153	82.75	-1.01	1.4E-04	Down
Nebl	chr2	17730506	17731424	169	124.17	-0.97	5.7E-05	Down
Irs2	chr8	11009140	11009975	-628	1391.99	-0.97	6.2E-15	Down
Plekha7	chr7	116307962	116308243	274	53.28	-0.96	3.7E-02	Down
Itgb3	chr11	104607854	104608505	179	2589.52	-0.95	5.9E-10	Down
Dhrs3	chr4	144891411	144891803	-1220	1181.11	-0.93	2.3E-11	Down
B4galnt3	chr6	120294288	120294779	364	71.89	-0.92	1.7E-02	Down
Angpt4	chr2	151911471	151911808	432	158.59	-0.91	3.1E-02	Down
Osbpl10	chr9	114978149	114978771	-25	107.71	-0.90	1.6E-02	Down
Tnfrsf1b	chr4	145246335	145246828	289	84.24	-0.88	1.7E-02	Down
Comp	chr8	70373951	70374830	842	330.84	-0.87	6.7E-04	Down
St3gal5	chr6	72097595	72098030	204	409.72	-0.85	3.0E-08	Down
Col3a1	chr1	45313187	45313472	1791	33156.18	-0.84	7.3E-07	Down
Ccn3	chr15	54745488	54745859	-237	8563.21	-0.83	1.6E-16	Down
Gli2	chr1	119052857	119053978	202	368.44	-0.83	1.3E-04	Down
Efhd1	chr1	87264395	87265128	408	2340.46	-0.80	5.0E-08	Down
Sh3pxd2a	chr19	47463686	47464143	497	7780.67	-0.80	2.2E-23	Down
Adra1d	chr2	131561118	131562720	578	764.40	-0.79	3.1E-04	Down
Eva1b	chr4	126148775	126149757	1263	644.40	-0.78	1.2E-13	Down
Nr4a2	chr2	57115697	57115932	-776	177.35	-0.77	4.0E-02	Down
Sncaip	chr18	52768175	52768587	237	197.18	-0.75	1.6E-02	Down
Fzd6	chr15	39006186	39006748	134	269.04	-0.75	5.0E-04	Down

Sdc1	chr12	8771472	8772159	419	530.98	-0.75	7.3E-04	Down
Rnd3	chr2	51147495	51148805	961	1413.21	-0.75	6.9E-06	Down
Plxna4	chr6	32588422	32588744	-391	1112.94	-0.74	1.2E-05	Down
Micall2	chr5	139735852	139736294	260	82.88	-0.74	3.1E-02	Down
Tmem132a	chr19	10869181	10869768	305	710.39	-0.74	4.9E-07	Down
Tinagl1	chr4	130175044	130175564	-182	3647.70	-0.72	4.3E-11	Down
Limch1	chr5	66745704	66746195	60	3666.67	-0.71	1.7E-09	Down
Agtrap	chr4	148087626	148088005	249	372.63	-0.71	4.9E-09	Down
Nhs	chrX	162158676	162159552	327	257.92	-0.71	2.8E-05	Down
Cpne2	chr8	94532202	94533514	-170	512.48	-0.70	5.0E-05	Down
Pde4b	chr4	102570538	102570778	598	481.85	-0.69	5.1E-03	Down
Ddi2	chr4	141722928	141723325	293	1631.08	-0.69	2.8E-16	Down
Bmpr1b	chr3	142168974	142169204	336	442.80	-0.68	2.6E-05	Down
Tmem136	chr9	43115968	43116633	270	359.75	-0.68	1.3E-06	Down
Mpzl1	chr1	165633965	165634392	363	171.45	-0.68	7.8E-04	Down
Sorl1	chr9	42123837	42124466	146	5207.04	-0.68	2.5E-08	Down
Fbxo30	chr10	11280937	11281860	68	3252.09	-0.68	8.5E-13	Down
Itga9	chr9	118606766	118607096	222	11772.16	-0.68	9.6E-08	Down
Alcam	chr16	52452494	52453479	95	671.16	-0.68	2.9E-04	Down
Aebp1	chr11	5862322	5862809	699	12268.87	-0.67	2.7E-07	Down
Tent5a	chr9	85327978	85328478	-1078	1241.64	-0.66	5.9E-04	Down
Pm20d2	chr4	33189055	33189781	319	106.61	-0.65	4.6E-02	Down
Ggta1	chr2	35461952	35462700	-877	552.40	-0.65	2.6E-09	Down
Adamtsl3	chr7	82335414	82335804	-85	1243.27	-0.65	5.2E-09	Down
Rassf3	chr10	121475742	121476253	253	3892.31	-0.64	1.3E-13	Down
Notch3	chr17	32166622	32166866	146	1811.13	-0.63	1.9E-10	Down
Hsph1	chr5	149635716	149636100	407	2139.38	-0.63	3.5E-02	Down
Myh10	chr11	68691261	68691694	-438	13933.29	-0.63	9.2E-10	Down
Prkd1	chr12	50648377	50649193	438	170.24	-0.63	7.0E-03	Down
Ndnf	chr6	65671788	65672159	362	99.27	-0.62	3.9E-02	Down
Atf3	chr1	191184554	191185163	-1525	876.94	-0.62	2.8E-02	Down
Amotl2	chr9	102717742	102718248	191	1514.09	-0.62	4.9E-02	Down
Ergic1	chr17	26561168	26561787	-35	1413.47	-0.60	3.3E-09	Down
Adam19	chr11	46055740	46057101	436	467.14	-0.60	3.8E-03	Down
Auts2	chr5	132542978	132543301	81	1423.38	-0.59	1.6E-06	Down
Sparc	chr11	55419088	55419900	586	28614.76	-0.59	5.4E-09	Down
Cmya5	chr13	93144330	93144554	282	298.80	-0.59	3.5E-03	Down
Arhgef10l	chr4	140647900	140648300	656	1747.65	-0.59	7.6E-09	Down
Fbn1	chr2	125504925	125506386	783	5919.12	-0.58	5.2E-05	Down
Endod1	chr9	14380538	14381299	324	2552.45	-0.58	2.4E-08	Down
Crybg3	chr16	59600595	59601316	92	670.13	-0.58	1.0E-04	Down

Slc41a3	chr6	90604928	90605255	211	266.73	-0.58	3.4E-02	Down
Gtf2ird1	chr5	134455776	134456354	197	247.37	-0.57	2.1E-03	Down
SEPT9	chr11	117266193	117267453	577	1950.38	-0.57	4.2E-05	Down
Pik3c2b	chr1	133046002	133046649	313	541.09	-0.56	7.6E-06	Down
Pgr	chr9	8901106	8901872	1656	315.60	-0.56	1.7E-02	Down
Faap20	chr4	155249735	155250509	156	157.16	-0.56	2.4E-02	Down
Mgat4a	chr1	37540680	37541013	170	1411.16	-0.56	8.5E-06	Down
Mast4	chr13	103333737	103335011	118	2207.86	-0.55	7.8E-08	Down
Vamp3	chr4	151057505	151057819	291	565.35	-0.55	1.4E-06	Down
Ksr1	chr11	79146060	79146357	265	435.48	-0.55	7.8E-04	Down
Nav2	chr7	49246747	49247241	805	2254.39	-0.54	1.5E-03	Down
Acvr1b	chr15	101175351	101175711	1459	383.84	-0.54	5.5E-04	Down
Smarcd3	chr5	24601408	24602597	0	1524.18	-0.54	1.2E-03	Down
Jun	chr4	95052574	95053120	-625	3692.21	-0.54	4.3E-02	Down
Fstl1	chr16	37777124	37777397	205	16194.20	-0.53	5.7E-08	Down
Fjx1	chr2	102450864	102451587	567	471.88	-0.53	7.8E-04	Down
Tagln2	chr1	172500300	172500586	197	3408.60	-0.53	2.8E-07	Down
Plekhg3	chr12	76533161	76533822	-69	3664.65	-0.53	3.1E-03	Down
Adamts2	chr11	50602091	50602790	355	3028.66	-0.53	4.7E-02	Down
Prdm11	chr2	93045733	93046096	251	199.07	-0.52	1.8E-02	Down
Tmem98	chr11	80810071	80810301	-180	324.33	-0.52	1.6E-02	Down
Aldh18a1	chr19	40588030	40588304	296	340.74	-0.51	6.6E-04	Down
Hr	chr14	70554079	70554515	241	2040.16	-0.51	3.1E-04	Down
Il4ra	chr7	125551308	125551826	-704	219.15	-0.51	2.7E-02	Down
Bcr	chr10	75060698	75061441	173	1299.81	-0.50	7.4E-05	Down
Lrp12	chr15	39942962	39943967	530	252.94	-0.50	2.7E-03	Down
Sulf2	chr2	166153973	166155330	634	2135.59	-0.49	1.1E-02	Down
Serinc5	chr13	92611194	92611476	197	743.90	-0.49	6.5E-04	Down
Fn1	chr1	71652794	71653061	307	58606.68	-0.49	1.6E-02	Down
Cxxc5	chr18	35829651	35830906	-36	528.93	-0.49	3.2E-03	Down
Loxl1	chr9	58312481	58313073	435	5128.27	-0.48	2.4E-05	Down
Prr13	chr15	102460527	102460979	1583	211.52	-0.47	3.6E-02	Down
Nxn	chr11	76398119	76399156	504	795.37	-0.47	6.1E-03	Down
Inpp5b	chr4	124741868	124742224	196	291.01	-0.46	7.8E-03	Down
Shb	chr4	45530907	45531229	-240	314.14	-0.46	4.2E-02	Down
Smad3	chr9	63757014	63758419	278	442.80	-0.46	1.6E-02	Down
Hacd4	chr4	88438530	88438908	207	455.01	-0.46	6.1E-03	Down
Col1a2	chr6	4505201	4505448	-294	30867.01	-0.46	2.9E-02	Down
Adamtsl4	chr3	95687332	95688157	173	452.73	-0.45	2.1E-02	Down
Zfp516	chr18	82909623	82909996	-1070	769.02	-0.45	1.6E-03	Down
Adgra3	chr5	50058417	50058899	338	772.89	-0.45	4.1E-03	Down

Arhgef2	chr3	88616207	88616710	251	3072.96	-0.45	1.9E-03	Down
Map3k4	chr17	12318189	12319018	57	572.18	-0.45	6.7E-04	Down
D1Ertd622e	chr1	97661479	97662194	-160	269.09	-0.45	3.1E-02	Down
Runx1t1	chr4	13742896	13744066	179	288.32	-0.45	7.2E-03	Down
Fbxo32	chr15	58214490	58214796	249	2788.77	-0.45	1.7E-04	Down
Npnt	chr3	132949332	132950000	148	15293.27	-0.45	9.2E-04	Down
Sec24d	chr3	123267154	123267726	-56	2397.68	-0.45	2.2E-05	Down
Ext1	chr15	53345798	53346532	18	2316.90	-0.44	1.5E-05	Down
Fam120c	chrX	151344434	151344812	400	272.06	-0.44	1.4E-02	Down
Synpo	chr18	60609188	60611570	-275	5939.21	-0.44	3.6E-03	Down
Large1	chr8	73354053	73354769	-871	1188.65	-0.44	3.6E-04	Down
Hacd1	chr2	14055785	14056184	51	606.92	-0.44	1.6E-04	Down
Irs1	chr1	82291766	82292057	-472	996.51	-0.44	1.1E-02	Down
Mtss2	chr8	110721187	110721897	66	2326.60	-0.44	3.3E-04	Down
Cav2	chr6	17281142	17281555	163	1660.81	-0.43	3.8E-06	Down
Loxl2	chr14	69609131	69609420	-201	1006.81	-0.43	2.0E-02	Down
Pkp4	chr2	59161247	59162015	-238	6740.33	-0.43	5.5E-05	Down
Znrf3	chr11	5444103	5444452	570	301.33	-0.43	3.6E-03	Down
Kremen1	chr11	5261317	5261596	154	455.13	-0.43	1.5E-02	Down
Ube2e3	chr2	78868923	78869894	-270	324.39	-0.42	6.7E-03	Down
Mfge8	chr7	79148649	79149010	231	7286.59	-0.42	1.3E-02	Down
Wdr1	chr5	38561158	38561542	349	3916.92	-0.42	4.5E-03	Down
Abca5	chr11	110337065	110337632	368	359.31	-0.42	3.0E-02	Down
Fosl2	chr5	32136197	32136442	-153	2182.18	-0.42	2.8E-02	Down
Grk5	chr19	60889839	60890097	219	884.85	-0.42	2.3E-03	Down
Ccdc9b	chr2	118763207	118763842	-863	1418.37	-0.41	2.6E-02	Down
Btg1	chr10	96617326	96618197	760	1560.38	-0.41	2.3E-04	Down
Ctsc	chr7	88278182	88278407	209	612.06	-0.41	4.4E-02	Down
Lrrfip1	chr1	91052634	91052998	-628	6028.08	-0.40	1.2E-05	Down
Vcl	chr14	20929652	20930617	736	9701.13	-0.40	1.8E-02	Down
Gja1	chr10	56378432	56378935	1383	2632.88	-0.40	5.5E-05	Down
Fam118a	chr15	85041722	85042119	-822	560.12	-0.40	4.3E-03	Down
Tpst1	chr5	130079486	130079924	335	658.36	-0.39	2.1E-02	Down
Pdzd2	chr15	12738929	12739956	450	1962.37	-0.39	4.5E-03	Down
Erg	chr16	95585396	95585852	969	1082.15	-0.39	6.3E-03	Down
Postn	chr3	54358964	54359273	-149	21753.97	-0.39	1.0E-03	Down
Ilk	chr7	105736680	105737269	249	4005.24	-0.39	2.8E-04	Down
Prdm6	chr18	53462600	53463006	-1743	1059.88	-0.39	4.6E-02	Down
Rnf11	chr4	109476180	109476559	136	1654.76	-0.38	1.3E-04	Down
Suclg2	chr6	95718255	95718774	332	707.79	-0.38	2.2E-03	Down
Uap1	chr1	170175313	170175545	-465	525.09	-0.38	1.9E-02	Down

Fam13c	chr10	70440606	70440979	-118	1530.39	-0.37	9.3E-04	Down
Slc48a1	chr15	97782829	97783199	-1351	1853.41	-0.37	1.9E-03	Down
Zmynd8	chr2	165858787	165859187	-814	610.93	-0.37	8.6E-03	Down
Mapk6	chr9	75409098	75409597	12	573.22	-0.36	1.2E-02	Down
Stimate	chr14	30825635	30825966	206	1841.83	-0.36	5.8E-03	Down
Emilin1	chr5	30912857	30913178	-769	3233.72	-0.36	1.6E-02	Down
Morf4l2	chrX	136741860	136742089	-365	1963.97	-0.35	2.3E-03	Down
Htra1	chr7	130936204	130936868	333	5553.44	-0.35	7.4E-03	Down
Glis2	chr16	4594066	4594319	-521	638.66	-0.35	2.9E-02	Down
Plin4	chr17	56108370	56108944	1145	7932.92	-0.35	3.2E-02	Down
Pcdh7	chr5	57717778	57720625	1120	12454.31	-0.35	5.6E-04	Down
Schip1	chr3	68493273	68494038	-526	1017.13	-0.35	1.1E-03	Down
Klf6	chr13	5861619	5861914	277	3948.47	-0.34	2.9E-03	Down
Fzd7	chr1	59482179	59485405	1645	1950.89	-0.34	2.7E-02	Down
Vldlr	chr19	27217097	27217323	190	2108.34	-0.34	4.7E-04	Down
Slc39a14	chr14	70350796	70351083	485	1055.57	-0.34	1.6E-02	Down
Soga1	chr2	157079352	157079611	-216	1917.59	-0.34	3.8E-03	Down
Reep5	chr18	34372631	34373422	389	2823.22	-0.34	1.0E-02	Down
Dnmt3a	chr12	3806522	3806901	-269	1709.02	-0.33	7.8E-04	Down
Stom	chr2	35336585	35336898	268	1839.95	-0.33	3.2E-03	Down
Szrd1	chr4	141139374	141139729	245	896.31	-0.33	9.7E-03	Down
Anxa11	chr14	25842294	25842579	281	2791.58	-0.33	1.4E-02	Down
Acadsb	chr7	131410732	131411033	281	1783.17	-0.33	8.0E-04	Down
Atxn1	chr13	45965201	45966267	-743	1128.21	-0.32	1.1E-02	Down
Fndc3b	chr3	27710031	27711266	-196	2796.83	-0.32	4.5E-02	Down
Flnb	chr14	7818058	7818381	262	2940.22	-0.32	2.1E-02	Down
Gnb4	chr3	32616252	32616620	154	1471.51	-0.32	2.8E-03	Down
Jmjd1c	chr10	67126723	67127343	-55	1316.83	-0.32	2.5E-03	Down
Lrp5	chr19	3685797	3686685	323	1577.84	-0.32	1.2E-03	Down
Furin	chr7	80400720	80401104	1900	1339.31	-0.32	2.9E-04	Down
Gas6	chr8	13493769	13494500	401	6082.85	-0.32	5.0E-02	Down
Wwtr1	chr3	57576142	57576710	-516	2185.68	-0.31	9.5E-03	Down
Qsox1	chr1	155812112	155813151	268	3394.64	-0.31	2.1E-02	Down
Col5a1	chr2	27886038	27887621	404	9206.82	-0.31	2.2E-02	Down
Erc1	chr6	119847852	119848447	1	980.73	-0.31	1.4E-02	Down
St3gal2	chr8	110919379	110919626	-363	2798.23	-0.30	2.6E-02	Down
Oxct1	chr15	4026344	4027328	408	6097.25	-0.30	1.3E-06	Down
Tbrg1	chr9	37657059	37657404	81	675.39	-0.30	2.6E-02	Down
Ube4b	chr4	149426095	149426556	306	1090.02	-0.30	1.1E-03	Down
Kmt5a	chr5	124445797	124446209	504	1348.52	-0.30	1.1E-02	Down
Inafm2	chr2	118745859	118746784	563	1290.63	-0.29	1.3E-03	Down

Ski	chr4	155221695	155222515	430	5676.30	-0.28	2.3E-02	Down
Pkn1	chr8	83697776	83698140	1221	2196.76	-0.28	2.1E-02	Down
Rin2	chr2	145785771	145786169	-146	1560.16	-0.28	2.9E-04	Down
Impad1	chr4	4792454	4792788	685	1964.34	-0.27	8.6E-03	Down
Zcchc24	chr14	25768163	25769042	470	2336.73	-0.27	3.6E-02	Down
Actg1	chr11	120348173	120348422	198	13707.75	-0.26	3.5E-02	Down
Twsg1	chr17	65950758	65951134	241	5403.79	-0.26	3.6E-03	Down
Plcb4	chr2	135659097	135660049	26	5339.29	-0.26	3.2E-03	Down
Phldb1	chr9	44733849	44735479	534	2548.28	-0.26	7.8E-03	Down
Aldh2	chr5	121593219	121593496	467	5589.60	-0.26	9.4E-03	Down
Ctdspl	chr9	118926153	118926443	-238	979.98	-0.25	3.4E-02	Down
Ccnd2	chr6	127151715	127152185	-902	4577.34	-0.25	4.5E-02	Down
Kdelr2	chr5	143403887	143404221	234	852.17	-0.25	3.2E-02	Down
Tlk2	chr11	105181675	105182355	488	647.17	-0.24	4.2E-02	Down
Mat2a	chr6	72439218	72439501	197	1609.44	-0.24	2.3E-02	Down
Uaca	chr9	60794458	60795235	404	2024.10	-0.24	2.1E-02	Down
Msrb3	chr10	120898402	120899429	56	7157.39	-0.24	2.3E-02	Down
Rab10	chr12	3309487	3309845	303	1690.10	-0.24	1.4E-02	Down
Bmpr1a	chr14	34502050	34502639	202	4037.95	-0.24	6.6E-03	Down
Dido1	chr2	180701496	180701875	417	1045.61	-0.23	9.0E-03	Down
Hdlbp	chr1	93478145	93478738	-64	4556.04	-0.23	1.5E-02	Down
Ppp1r9b	chr11	94989385	94989893	-1573	3248.50	-0.23	5.3E-03	Down
Cav1	chr6	17306400	17308334	-273	8584.30	-0.21	4.2E-02	Down
Ppp1cb	chr5	32459411	32459697	584	6486.45	-0.21	2.7E-02	Down
Mef2a	chr7	67372451	67373172	47	4000.83	-0.20	1.4E-02	Down
Mprip	chr11	59662673	59663023	341	6535.24	0.21	7.2E-03	Up
Lix1l	chr3	96601133	96602129	498	1489.07	0.24	2.0E-02	Up
Celf1	chr2	90940499	90940997	351	1670.70	0.26	6.1E-04	Up
Trip10	chr17	57250261	57250499	929	1079.67	0.26	3.0E-03	Up
Ankrd12	chr17	66077166	66077552	-313	1725.07	0.27	1.7E-02	Up
Arhgap21	chr2	20966962	20967358	561	1090.18	0.28	3.9E-02	Up
Prdx6	chr1	161250751	161251073	298	719.01	0.28	1.5E-02	Up
Arl6ip5	chr6	97210822	97211620	429	1740.55	0.29	4.3E-02	Up
Pbx1	chr1	168431338	168431596	702	2496.93	0.29	2.0E-02	Up
Arhgap10	chr8	77517265	77518289	130	473.69	0.29	4.3E-02	Up
Igf1r	chr7	67951383	67951762	-685	2809.56	0.29	4.6E-02	Up
Mllt6	chr11	97663841	97664256	636	2063.24	0.30	1.5E-04	Up
Zdhhc1	chr8	105496721	105497046	19	468.13	0.30	4.5E-02	Up
Tgfbr3	chr5	107288951	107289567	370	1840.63	0.30	2.0E-02	Up
Maco1	chr4	134852678	134853044	484	941.70	0.30	1.9E-02	Up
Acsl4	chrX	142390053	142390414	302	366.31	0.30	5.0E-02	Up

Efr3a	chr15	65787108	65787600	334	1565.60	0.31	2.8E-03	Up
Csnk1e	chr15	79441709	79441958	4	651.22	0.31	7.4E-03	Up
Prnp	chr2	131909937	131910346	213	1537.62	0.31	4.9E-03	Up
Limd1	chr9	123479080	123479518	598	750.36	0.31	1.4E-02	Up
Eid1	chr2	125673166	125673669	317	1498.20	0.32	4.3E-03	Up
Tes	chr6	17065206	17065956	432	1645.82	0.32	1.1E-02	Up
Cdk6	chr5	3342095	3343043	-1324	560.14	0.32	2.6E-02	Up
Dnajc1	chr2	18392311	18392752	299	1149.15	0.32	3.5E-03	Up
Fgfr1	chr8	25518871	25519931	642	4064.90	0.32	1.3E-03	Up
Luzp1	chr4	136469783	136470810	535	1759.03	0.32	5.4E-03	Up
Ppp1r12c	chr7	4502080	4502359	-539	4302.98	0.33	3.6E-02	Up
Foxp1	chr6	99520014	99522358	-193	2543.58	0.33	5.9E-03	Up
Ltbp1	chr17	75005821	75006417	590	11684.25	0.33	4.2E-03	Up
Sesn3	chr9	14276340	14276973	355	794.33	0.34	5.8E-03	Up
Nr2f2	chr7	70360032	70361647	-246	1425.87	0.34	1.1E-02	Up
Cnst	chr1	179546483	179546876	150	552.79	0.34	4.9E-02	Up
Notch2	chr3	98013570	98013959	226	3073.66	0.34	5.1E-03	Up
Ano1	chr7	144738093	144738423	334	1494.50	0.35	1.3E-02	Up
Fam53a	chr5	33628871	33629555	422	546.33	0.35	8.4E-03	Up
Bcl2	chr1	106713739	106714191	325	1156.05	0.36	3.5E-03	Up
Ccdc85b	chr19	5456436	5457506	592	1770.54	0.36	3.3E-03	Up
Rgs7bp	chr13	105054163	105054859	419	1505.48	0.37	7.3E-04	Up
Cds2	chr2	132263304	132263643	325	5099.16	0.37	5.3E-03	Up
Mmd	chr11	90249531	90250009	294	1074.42	0.37	4.5E-02	Up
Mark4	chr7	19459069	19459519	-451	482.19	0.37	2.0E-03	Up
Inppl1	chr7	101837619	101837967	31	1071.21	0.37	2.6E-02	Up
App	chr16	85173211	85173939	132	9454.66	0.38	2.6E-04	Up
Rnd2	chr11	101468164	101468632	223	384.41	0.38	4.9E-02	Up
Mark1	chr1	184999156	184999636	153	1981.56	0.39	1.5E-02	Up
Rev1	chr1	38129417	38129664	126	384.10	0.39	4.2E-02	Up
Bahd1	chr2	118901449	118901697	-42	303.49	0.39	3.0E-02	Up
Foxc2	chr8	121116329	121118400	1193	992.15	0.39	1.6E-02	Up
Ell2	chr13	75707600	75708424	528	733.74	0.40	4.9E-02	Up
Vim	chr2	13573353	13575097	-86	51911.13	0.40	2.8E-02	Up
Ank	chr15	27466208	27467338	96	1238.97	0.40	3.6E-03	Up
Mcam	chr9	44135063	44135320	533	9509.39	0.41	3.8E-02	Up
Maf	chr8	115706319	115707547	-39	1228.07	0.41	6.7E-05	Up
Mthfr	chr4	148041241	148042005	434	521.54	0.41	2.4E-02	Up
Psme4	chr11	30771790	30772236	238	1182.50	0.42	9.8E-07	Up
Cdk19	chr10	40349695	40349994	536	530.74	0.42	2.4E-02	Up
Stim2	chr5	53998486	53999132	286	430.29	0.43	3.4E-02	Up

Cst3	chr2	148874857	148875289	439	8469.72	0.43	5.6E-04	Up
Fam124a	chr14	62555517	62556152	97	495.94	0.43	2.5E-02	Up
Smad1	chr8	79398227	79399422	604	443.49	0.43	1.1E-02	Up
C2cd2	chr16	97921903	97922618	373	1148.06	0.43	5.2E-03	Up
Pxdc1	chr13	34652208	34653341	-93	627.76	0.44	4.8E-02	Up
Pip4k2a	chr2	18997374	18998067	401	665.49	0.44	3.5E-04	Up
Nr1d2	chr14	18238671	18239010	266	2459.32	0.44	7.6E-06	Up
Mapk7	chr11	61493325	61494199	499	252.66	0.44	1.5E-02	Up
Dzip1	chr14	118924809	118925424	354	304.40	0.45	6.6E-03	Up
Clcn6	chr4	148038436	148038776	207	270.11	0.45	1.6E-02	Up
Pold3	chr7	100121184	100121487	165	187.63	0.45	3.5E-02	Up
Spats2l	chr1	57774735	57775647	330	762.86	0.45	2.9E-03	Up
Socs2	chr10	95415575	95417461	-306	568.85	0.45	8.4E-04	Up
Tmtc1	chr6	148442790	148444747	584	4200.01	0.45	3.7E-05	Up
Gm13889	chr2	93956464	93957065	336	3310.94	0.46	2.4E-04	Up
Hmga1	chr17	27555289	27555540	-1160	210.11	0.46	3.1E-02	Up
Plekhh2	chr17	84511881	84512430	260	386.47	0.47	5.1E-04	Up
Gcnt2	chr13	40917377	40917848	-22	1092.49	0.47	1.9E-04	Up
Phlda1	chr10	111506634	111507140	601	217.86	0.47	4.9E-02	Up
Pde1a	chr2	80038323	80038599	441	424.75	0.48	3.7E-04	Up
Nes	chr3	87971097	87971518	214	6026.37	0.48	8.9E-04	Up
Sulf1	chr1	12691949	12692322	-295	3969.91	0.49	1.1E-03	Up
Slc6a6	chr6	91684120	91684651	318	2799.28	0.49	8.3E-07	Up
Klhl7	chr5	24100722	24101087	314	673.13	0.49	1.1E-06	Up
Pde4dip	chr3	97767540	97767927	281	6312.74	0.49	6.5E-06	Up
Bahcc1	chr11	120233540	120235247	1446	555.89	0.50	4.8E-05	Up
Rell1	chr5	63968261	63968816	359	251.45	0.50	2.0E-02	Up
Cd109	chr9	78615507	78616047	231	788.66	0.50	5.5E-03	Up
Csrp2	chr10	110919764	110920767	89	12001.25	0.50	1.1E-06	Up
Mxra7	chr11	116826733	116828014	673	1502.79	0.50	2.8E-04	Up
Efhd2	chr4	141874526	141874837	239	1520.15	0.51	3.2E-07	Up
Flt3l	chr7	45135780	45136045	520	155.08	0.51	4.5E-02	Up
Npr3	chr15	11904364	11905596	694	536.97	0.51	8.6E-03	Up
Map3k5	chr10	19933621	19934870	-281	994.03	0.51	2.9E-04	Up
Flnc	chr6	29432963	29433498	-26	5490.48	0.52	1.4E-02	Up
Xylt1	chr7	117380357	117381772	85	556.46	0.52	6.8E-03	Up
Yae1d1	chr13	17993062	17993440	100	662.30	0.52	1.1E-03	Up
Hoxb6	chr11	96299097	96299756	352	157.54	0.52	3.1E-02	Up
Mecom	chr3	30012927	30013191	145	562.61	0.53	3.3E-05	Up
Usp31	chr7	121706549	121707027	654	331.87	0.53	3.0E-03	Up
Asap1	chr15	64313162	64313838	-827	990.67	0.53	3.6E-06	Up

Olfml2b	chr1	170644416	170644773	62	2447.52	0.54	1.1E-06	Up
Tef	chr15	81802833	81803137	312	3341.32	0.54	1.4E-05	Up
Lonrf1	chr8	36248016	36249352	675	388.70	0.55	1.7E-02	Up
Zc3hav1	chr6	38354290	38354550	183	445.82	0.55	1.2E-04	Up
Inhbb	chr1	119420012	119422554	965	167.07	0.55	3.6E-02	Up
Rap2b	chr3	61363176	61363595	-1122	222.97	0.56	4.7E-02	Up
Palld	chr8	61590631	61591191	228	2971.90	0.56	8.8E-04	Up
Zswim6	chr13	107890922	107891448	-1121	272.31	0.56	1.9E-03	Up
Mrtfa	chr15	81189963	81190444	533	402.11	0.56	5.8E-03	Up
Tgfb2	chr1	186705118	186705652	604	1691.29	0.56	1.1E-04	Up
Lpin2	chr17	71183672	71184118	-83	156.06	0.57	3.6E-02	Up
Zfp346	chr13	55105363	55105679	212	182.68	0.57	1.2E-03	Up
Ikzf2	chr1	69687065	69687791	-1468	131.10	0.57	1.1E-02	Up
Satb1	chr17	51832657	51833281	321	361.92	0.57	5.5E-06	Up
Nlgn2	chr11	69836656	69837994	459	647.16	0.58	3.3E-04	Up
Klf10	chr15	38299459	38300664	-161	287.32	0.59	3.4E-02	Up
Rbm38	chr2	173022143	173022429	384	140.20	0.59	3.7E-02	Up
Trim2	chr3	84220511	84220865	189	341.13	0.59	1.6E-02	Up
Vcan	chr13	89742070	89742574	190	1632.57	0.59	2.1E-05	Up
Fam102b	chr3	109027020	109027581	307	722.88	0.59	5.7E-05	Up
Zfp462	chr4	54946173	54946419	-1649	122.06	0.59	1.2E-02	Up
Pdgfc	chr3	81036423	81037146	368	442.39	0.59	5.5E-06	Up
Podxl2	chr6	88874589	88874826	-111	376.80	0.59	2.7E-04	Up
Tmem200b	chr4	131921263	131922009	-135	550.42	0.61	1.1E-07	Up
Sned1	chr1	93235792	93236522	260	464.25	0.61	2.8E-03	Up
Tsc22d3	chrX	140599211	140599550	1142	447.97	0.61	2.0E-02	Up
Sptb	chr12	76710088	76710313	347	224.44	0.61	2.9E-02	Up
Hhipl1	chr12	108306325	108306713	249	125.23	0.62	1.3E-02	Up
Trnp1	chr4	133497618	133498057	713	304.68	0.62	1.4E-02	Up
Cpeb1	chr7	81454406	81455032	39	310.91	0.62	1.5E-03	Up
Cys1	chr12	24680602	24681357	816	569.66	0.63	3.7E-07	Up
Plaat1	chr16	29209906	29210337	13	101.40	0.63	4.1E-02	Up
Coq10b	chr1	55052876	55053353	344	746.70	0.63	8.5E-05	Up
Fibin	chr2	110362366	110363010	305	2468.41	0.64	3.1E-05	Up
Ntn4	chr10	93640805	93642252	479	1378.44	0.65	1.0E-04	Up
Id3	chr4	136143910	136144349	307	915.77	0.65	1.4E-07	Up
Cdc42ep1	chr15	78842461	78843410	288	280.29	0.65	1.0E-02	Up
Dennd5b	chr6	149100593	149101602	583	593.71	0.66	2.0E-05	Up
Tspan4	chr7	141474927	141475791	123	487.98	0.66	4.3E-05	Up
Pcgf5	chr19	36348116	36349305	381	907.19	0.68	7.1E-09	Up
1700017B05Rik	chr9	57261686	57262521	496	352.67	0.70	5.1E-07	Up

Sox12	chr2	152396634	152397598	930	270.93	0.70	1.7E-04	Up
Ablim1	chr19	57198787	57199074	-1299	2243.10	0.72	5.7E-08	Up
Smad7	chr18	75367459	75368198	463	1119.48	0.72	1.2E-03	Up
Sdc3	chr4	130792048	130792345	-341	821.52	0.72	2.6E-05	Up
Etv5	chr16	22439320	22439582	119	203.19	0.73	1.1E-03	Up
Ss18l1	chr2	180042852	180043303	594	96.28	0.73	1.7E-02	Up
Maml3	chr3	52104685	52105256	36	174.72	0.73	2.7E-04	Up
Cnksr3	chr10	7211639	7212052	392	667.53	0.73	1.0E-04	Up
Hlf	chr11	90389975	90390298	781	824.40	0.74	8.6E-05	Up
Ptpru	chr4	131836282	131837154	1560	131.38	0.74	5.0E-03	Up
Spata13	chr14	60634095	60635203	-56	798.54	0.74	2.2E-10	Up
Wee1	chr7	110122199	110122961	521	218.81	0.75	1.1E-03	Up
Per2	chr1	91458521	91458818	659	1482.11	0.75	1.0E-05	Up
Pde7a	chr3	19311394	19311754	-252	310.55	0.75	2.9E-06	Up
Epas1	chr17	86753583	86754171	13	6133.94	0.76	4.3E-08	Up
Foxf1	chr8	121084225	121084903	178	281.84	0.76	7.8E-04	Up
Ntrk3	chr7	78578759	78579385	-1234	4352.59	0.78	3.0E-10	Up
Olfm1	chr2	28193039	28193393	123	482.25	0.78	4.1E-04	Up
Bhlhe41	chr6	145865570	145866783	-756	949.74	0.79	2.0E-07	Up
Unc5b	chr10	60830630	60831420	556	740.41	0.80	3.0E-06	Up
Sox4	chr13	28952080	28953132	1093	513.96	0.80	6.6E-10	Up
Arhgap42	chr9	9238729	9239299	-1	101.10	0.83	5.5E-04	Up
Trib2	chr12	15815544	15816001	1013	334.00	0.83	4.4E-07	Up
Baz1a	chr12	54985171	54985761	870	133.99	0.84	3.9E-02	Up
Pde5a	chr3	122729610	122730439	866	4505.51	0.84	4.9E-27	Up
Ier5l	chr2	30472856	30473954	794	353.87	0.85	1.4E-07	Up
Dipk1a	chr5	107987237	107987473	-278	144.15	0.86	8.0E-05	Up
Zfp503	chr14	21988738	21989653	406	401.33	0.86	1.4E-07	Up
Plk2	chr13	110394505	110394915	-334	87.99	0.87	4.5E-03	Up
Cpxm1	chr2	130397521	130397782	-22	365.30	0.87	2.2E-02	Up
Tril	chr6	53820590	53820826	117	187.90	0.89	1.7E-04	Up
Bok	chr1	93685593	93685921	182	74.44	0.90	1.4E-02	Up
Lratd2	chr15	60824143	60824890	564	156.22	0.90	2.5E-03	Up
Oasl2	chr5	114897379	114897913	712	102.35	0.93	9.8E-03	Up
Fbxl7	chr15	26895052	26895889	94	208.94	0.93	1.6E-06	Up
Zfp697	chr3	98382952	98383244	617	89.85	0.94	2.8E-04	Up
H2-Q4	chr17	35379666	35379990	211	444.36	0.97	8.7E-04	Up
Podn	chr4	108031868	108032257	28	900.14	0.97	7.4E-07	Up
Tnfrsf21	chr17	43016683	43017750	661	121.15	0.99	1.7E-02	Up
Sorbs1	chr19	40513446	40513687	170	1577.25	0.99	2.8E-23	Up
Cd44	chr2	102902226	102902490	-693	946.52	0.99	1.3E-14	Up

Hes1	chr16	30062900	30064327	-1744	194.55	1.00	4.6E-07	Up
Bend7	chr2	4717774	4718444	278	47.87	1.02	2.0E-02	Up
Mthfd1l	chr10	3973258	3973759	433	113.99	1.08	1.0E-05	Up
Lbh	chr17	72917633	72917987	-495	3720.31	1.08	1.2E-19	Up
Plat	chr8	22758038	22758477	535	942.96	1.23	1.2E-26	Up
Spaar	chr4	43728497	43729294	-1139	55.23	1.29	2.0E-02	Up
Bbc3	chr7	16309275	16309988	48	80.19	1.29	1.5E-07	Up
Nxph3	chr11	95513984	95514408	369	31.21	1.30	4.5E-03	Up
Bhlhe40	chr6	108659170	108659449	-1320	565.48	1.34	1.5E-17	Up
H2-Ab1	chr17	34264472	34265022	1520	233.45	1.34	7.3E-04	Up
Adamts14	chr10	61273264	61273810	-118	230.11	1.35	6.0E-11	Up
Prag1	chr8	36094673	36095244	130	151.41	1.36	2.3E-07	Up
Prr5l	chr2	101796479	101796968	984	161.06	1.43	2.2E-13	Up
Mex3b	chr7	82865635	82866067	-1482	48.98	1.43	1.2E-03	Up
Pde1b	chr15	103502863	103503268	31	60.70	1.46	7.5E-06	Up
Coro6	chr11	77464384	77464811	686	275.04	1.51	3.9E-14	Up
Slc8a3	chr12	81332721	81333018	311	85.53	1.70	4.1E-06	Up
Kcnc4	chr3	107459167	107459857	-614	806.26	1.74	2.5E-11	Up
Cry1	chr10	85184395	85185043	335	110.88	1.77	1.2E-10	Up
Sap30	chr8	57486813	57487769	569	21.41	1.85	8.6E-03	Up
Wnk2	chr13	49147112	49147944	800	105.88	1.94	3.7E-09	Up
Gpr176	chr2	118372758	118374401	-160	30.02	1.94	4.6E-03	Up
4930426L09Rik	chr2	18998367	18998744	236	26.35	1.97	6.5E-05	Up
Slc6a17	chr3	107517577	107517949	255	369.82	2.00	6.3E-33	Up
Wasf1	chr10	40883501	40884535	484	36.39	2.03	1.9E-07	Up
Masp1	chr16	23518752	23519571	1654	35.74	2.05	1.0E-05	Up
Slco5a1	chr1	12991775	12992927	-1216	14.29	2.35	1.9E-03	Up

Supplemental Table 4. qPCR primers used in this study.

Targets	Forward primer	Reverse primer
Gapdh (mouse)	CTTTGTCAAGCTCATTTCCTGG	TCTTGCTCAGTGTCCTTGC
Prdm16 (mouse)	AGTCGGACAACCATGCACTT	GATCTCAGGCCGTTTGTCCA
Adra1a (mouse)	TCCAACCATTAAGATCCACACC	CCTAGTGTCATCCCTTTTCCTG
Adra1b (mouse)	TTCATCTTATGTTGGCTCCCC	ACACTACCTTGAATACGGCG
Adra1d (mouse)	TGTCCCTAAATGTTCCCCAAG	CTATAAAGCACCCCACCTCTG
Bmal1 (mouse)	CAACCCATACACAGAAGCAAAC	CATCTGCTGCCCTGAGAATTA
Npas2 (mouse)	ACAAGGGGACATTTAGCGGG	CTCTCTCTTGGCGAGCAGC
Per1 (mouse)	TCGAAACCAGGACACCTTCTCT	GGGCACCCCGAAACACA
Per2 (mouse)	AATCTTCCAACACTCACCCC	CCTTCAGGGTCCTTATCAGTTC
Per3 (mouse)	GGCTGCTTTGATCCTGAATTCT	GAACGCCCTCACGTCTTGAG
Cry1 (mouse)	TCGCCGGCTCTTCCAA	TCAAGACACTGAAGCAAAAATCG
Cry2 (mouse)	CCCACGGCCCATCGT	TGCTTCATTCGTTCAATGTTGAG
Adra1d-ChIP	GTCACCAGGCCGTGAAG	CAGGACACGCTTGGAGAG
Bmal1-ChIP	CTTGCCTGGTCAACCCTTCT	CAAGACAGGCCATAGGGACG
Npas2-ChIP	CCCGCTCGGAGGTAAGAG	AGCCACGCGCACGTATT
Per2-ChIP	GGTTCCTCAATGAAGATGCTG	CAGGTGAATGGAAGTCCCG
Cry1-ChIP	GGCTCATAACCGACACCTG	TCCATTCATCTGCCAGCTG
Cry2-ChIP	TCCCCTCACCTCCATCG	GCTGCGTCTACATCCTCG
Gapdh (rat)	TCCAGTATGACTCTACCCACG	CACGACATACTCAGCACCAG
Prdm16 (rat)	AGAAGCACGAACACGAAGGT	ATCTCGCTGTTGGCGATGAA
Myh11 (rat)	CCAGATAGATGCAGGTCGGG	ACTGGAAGGTCAGGCAGTTG
Acta2 (rat)	CATCACCAACTGGGACGACA	TCCGTTAGCAAGGTCGGATG
Cnn1 (rat)	TCCGCACACTTTAACCGAG	ATCCATGAAGTTGCTCCCG
Tagln (rat)	GCGTGATTCTGAGCAAGTTG	CGTGACTCCATAATCCTCAGC
Bmal1 (rat)	ACCAACCCATACACAGAAGC	GACAGATTCGGAGACAAAGAGG
Npas2 (rat)	TAGGGTGGAAAGCAGAGGGC	AGAGTTTCTATGCAGTTTTGCCTTA
Adra1d (rat)	CTGCTCAAGTTTTCCCGC	AAGATGACCTTGAAGACACCC
