# Supplementary material for: Vascular smooth muscle cell PRDM16 regulates circadian variation in blood pressure
Source: J Clin Invest. 2024 Dec 3;135(3):e183409. doi: 10.1172/JCI183409 (PMC11785921; doi:10.1172/JCI183409)

Full unedited blot for Figure 4B

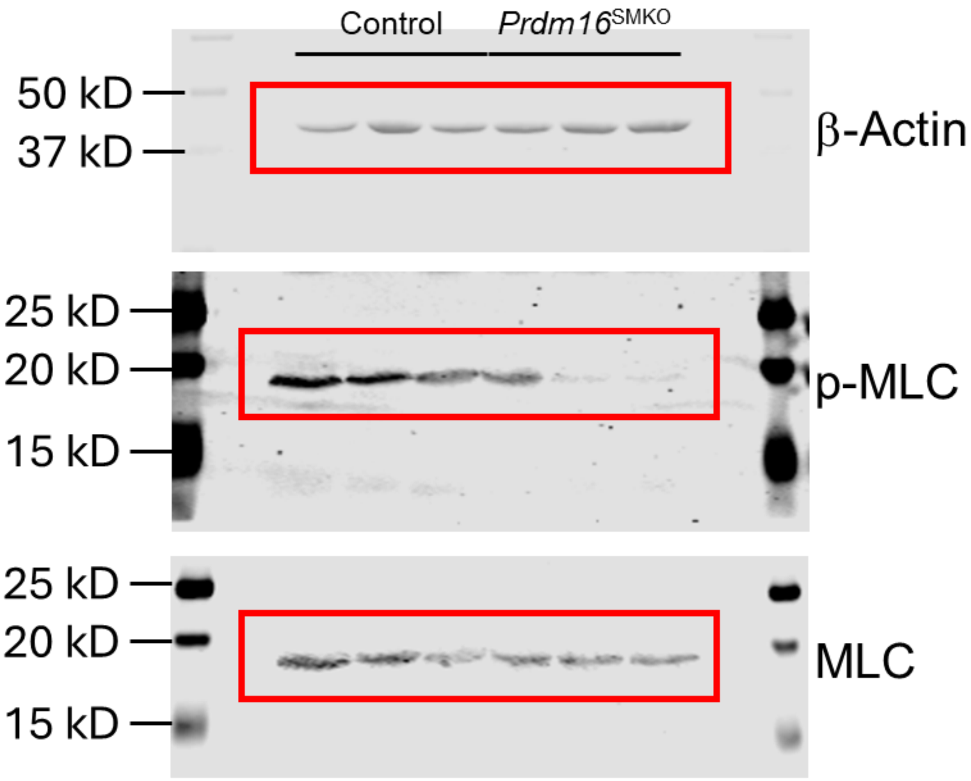

Full unedited blot for Figure 4D

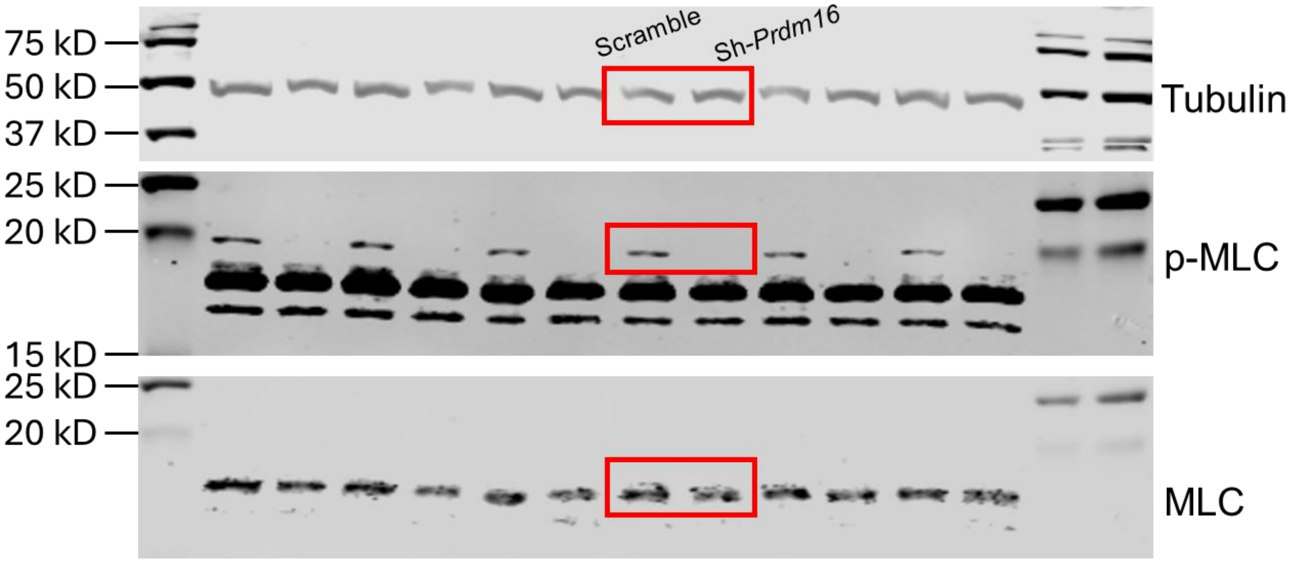

Full unedited blot for Figure 5C

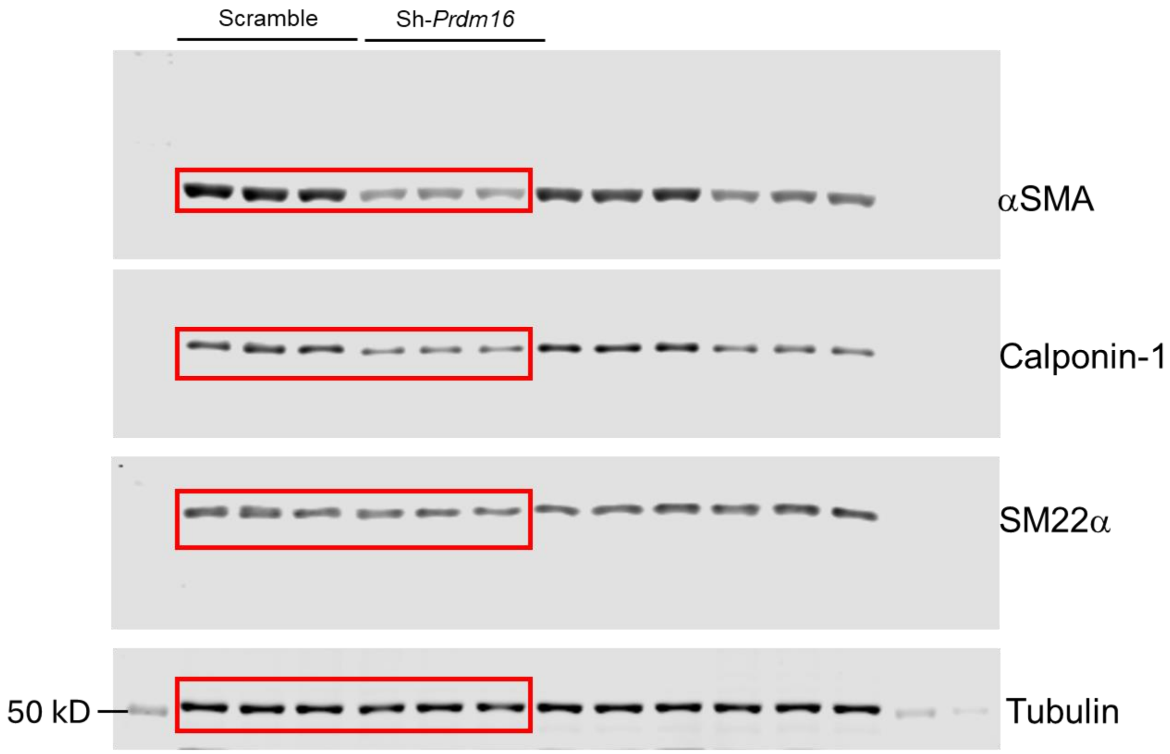

Full unedited blot for Figure 5E

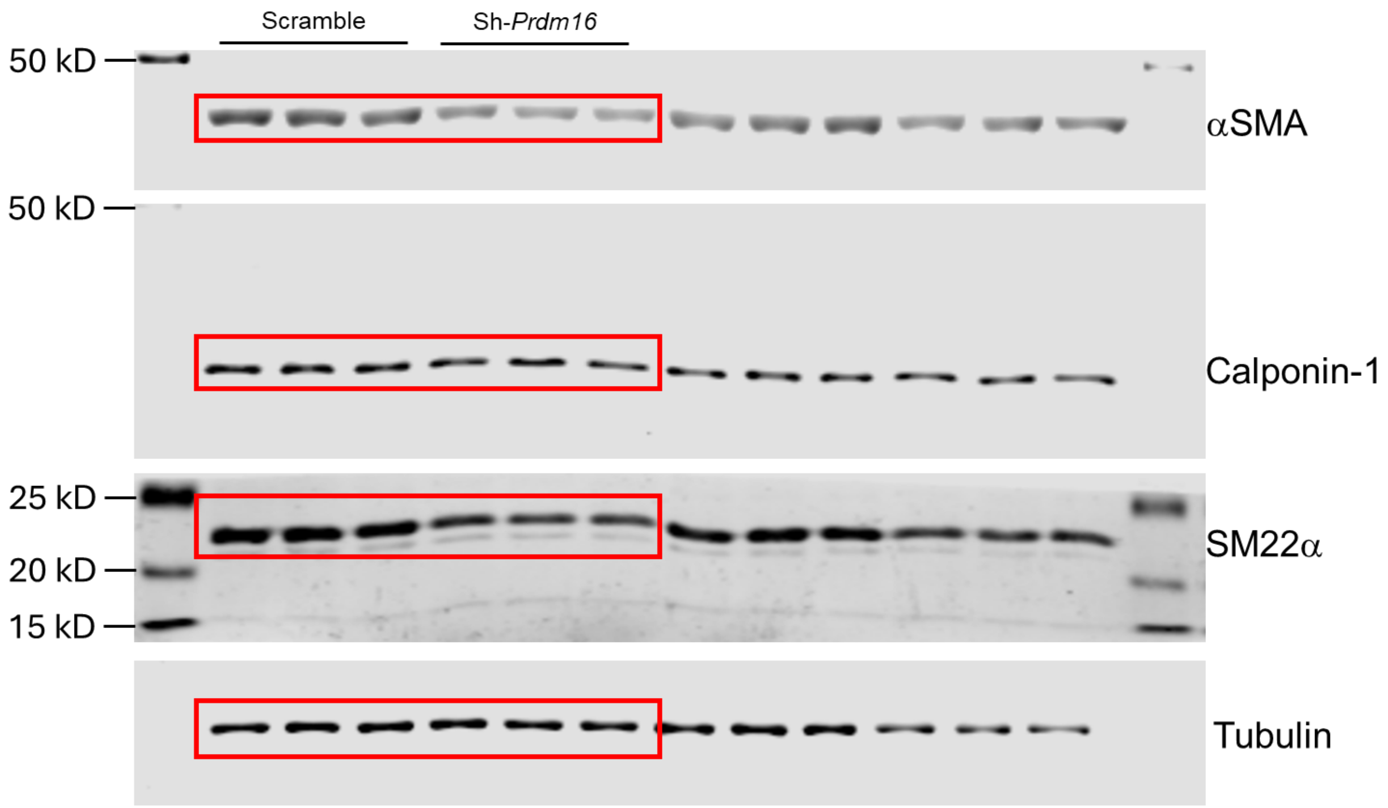

Full unedited blot for Figure 5F

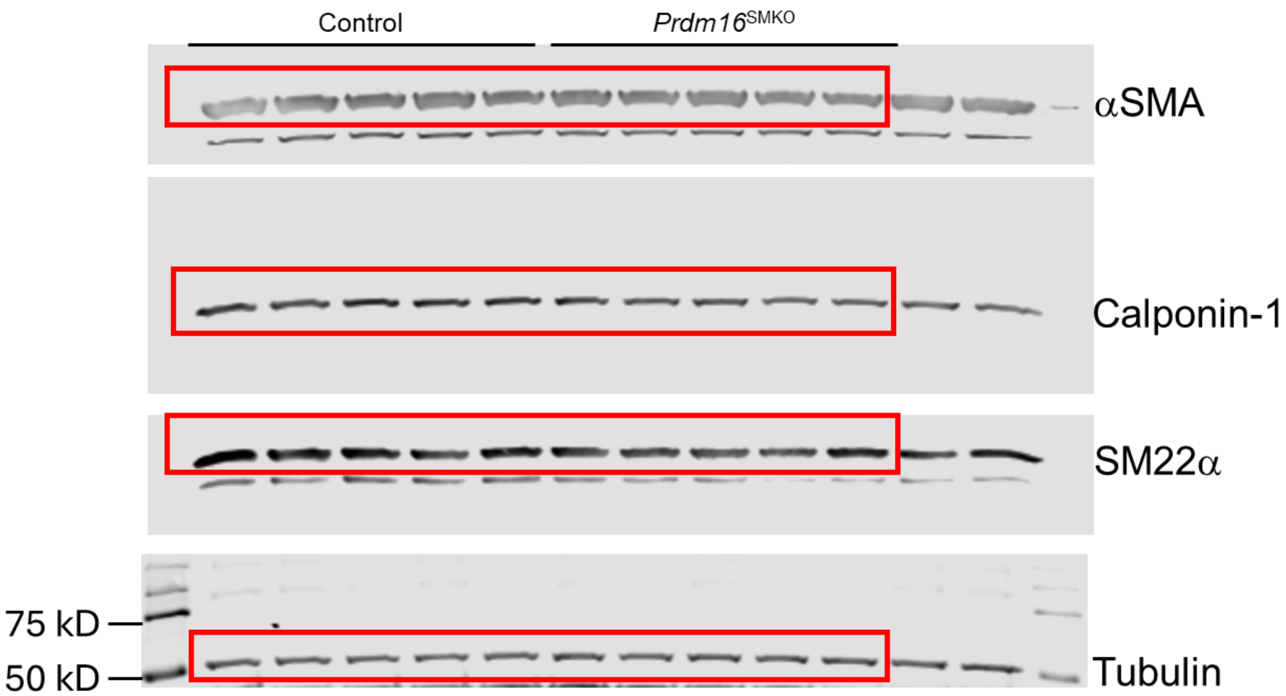

Supplement: Unedited blot and gel images [file jci-135-183409-s112.pdf]
